# Stress-mediated polysorbate 20 degradation and its potential impact on therapeutic proteins

**DOI:** 10.1007/s11095-024-03700-7

**Published:** 2024-05-13

**Authors:** Baikuntha Aryal, Mari Lehtimaki, V. Ashutosh Rao

**Affiliations:** Laboratory of Applied Biochemistry, Division of Biotechnology Research and Review III, Office of Biotechnology Products, Office of Pharmaceutical Quality, Center for Drug Evaluation and Research, Food and Drug Administrations, Silver Spring, MD 20993 USA

**Keywords:** Polysorbate, esterase, lipase, free fatty acid, biological activity

## Abstract

**Purpose:**

Polysorbates are the most commonly used surfactants in formulations to stabilize therapeutic proteins against interfacial stresses. Polysorbates can undergo oxidative or enzyme-mediated hydrolytic degradation to produce free fatty acids (FFAs) and subvisible particles in formulations. To determine which product related variables contribute to PS20 degradation, we investigated the effects of storage temperature, formulation, pH, presence of hydrolytic enzymes, and specific fatty acid composition on different grades of PS20 in relation to their PS20 degradation profile and consequently the quality of protein drug products.

**Methods:**

Bevacizumab and T-DM1 were reformulated in the freshly prepared therapeutic protein formulations containing either compendial PS20 or non-compendial PS20 with high % lauric acid and spiked with exogenous esterase or lipase. The release of FFAs and formation of particles were monitored at 4°C and 37°C. Protein quality was assessed for secondary structures, purity, and biological activity.

**Results:**

Hydrolytic release of FFAs and formation of subvisible particles were found to be dependent on grades of PS20, types of enzymes used, incubation temperature, and pH. Esterase- or lipase-mediated degradation of PS20 and formation of subvisible particles in drug formulation showed no significant impact on the biological activity and stability of therapeutic proteins against degradation or aggregation.

**Conclusions:**

Our study suggests that degradation of PS20 and formation of FFA particles depend on the fatty acid composition of PS20, types of hydrolytic enzymes, pH, and temperature. The presence of FFA subvisible particles showed no significant impact on the purity and biological activity of the therapeutic proteins under the tested conditions.

**Supplementary Information:**

The online version contains supplementary material available at 10.1007/s11095-024-03700-7.

## Introduction

Polysorbates are nonionic amphipathic surfactants used in biotherapeutic formulations to protect biologics against interfacial stresses during manufacturing, shipping, and storage. Polysorbate 20 (PS20) and polysorbate 80 (PS80) are the most commonly used surfactants in monoclonal antibody formulations to increase protein stability and to reduce aggregation (1). Polysorbates are composed of a common sorbitan ring, ethylene oxide polymers attached at three different hydroxyl positions and fatty acid moieties attached to the ethylene oxide oxygen through an ester linkage. The number of ethylene oxide subunits varies at each position, but the total number of polyethylene oxide moieties equals 20 and is constant for all polysorbates (2). Fatty acid composition in PS20 and PS80 includes saturated fatty acid such as caproic acid (C6), caprylic acid (C8), capric acid (C10), lauric acid (C12), myristic acid (C14), palmitic acid (C16), stearic acid (C18) and unsaturated fatty acids such as palmitoleic acid (C16:1), oleic acid (C18:1), linoleic acid (C18:2) and linoleic acid, C18:3) with 1, 2 and 3 carbon–carbon double bonds respectively. More than one type of saturated and unsaturated fatty acids with carbon lengths ranging from 6 to 18 are esterified to polyoxyethylene sorbitan during the synthesis of polysorbate; therefore, there is heterogeneity in fatty acid composition in polysorbate, and the degree of heterogeneity may vary from lot to lot (3, 4). In addition, residual free fatty acid (FFA), polyoxyethylene (POE) head group, sorbitan POE, number of ethylene oxide units, and isosorbide POE fatty acid ester during synthesis of polysorbates (mono-, di- or triesters of fatty acids) contribute to lot-to-lot variability for polysorbates (5, 6). The European Pharmacopoeia (EP) and the United States Pharmacopoeia National Formulary (USP-NF) specify the heterogeneity in fatty acid esters of each polyoxyethylene sorbitan within the predefined acceptable range (4). Lauric acid, a C12 fatty acid, and oleic acid, a C18:1 fatty acid, are the major constituents of the fatty acid mixture in PS20 NF and PS80 NF, respectively. In addition to compendial grade, super refined and high purity grades of PS20 and PS80 with reduced heterogeneity in fatty acid composition are commercially available (4).

Polysorbates are known to undergo chemical or enzymatic degradation in biopharmaceutical formulations over time during storage to form free fatty acids (FFAs) resulting in the formation of visible or subvisible particles when the FFA concentration exceed their solubility limits in aqueous solution (7, 8). The traces of residual hydrolytic enzymes such as esterases or lipases that co-purified with therapeutic proteins as host cell proteins (HCPs) impurities during the biotechnological drug substance manufacturing process degrades polysorbate to produce FFAs and reactive aldehydes and ketones (9–11). Lipases and esterases can have hydrolytic activity on PS20 and PS80 when present in the purified therapeutic protein drugs as low as 1 ppm under normal storage conditions (12, 13). Phospholipase B-like 2 (PLBD2) protein was initially proposed to be a potential HCP responsible for degrading polysorbates in drug products purified from Chinese hamster ovary (CHO) cells, but a recent study does not support PLBD2’s role in polysorbate degradation (10, 14). Additionally, there have been efforts to reduce the number of hydrolytic enzymes in HCP impurities by genetic knockout of lipoprotein lipase (LPL) in CHO cells to improve the stability of polysorbates in drug formulations (15). In addition to enzymatic hydrolysis, degradation of polysorbate can be catalyzed by oxidative impurities including temperature, light, and traces of metal ions present in the formulation (1, 9, 16, 17). The protein concentration in the drug product formulation can also influence polysorbate degradation and subsequent particle formation (16).

Polysorbate degradation and the formation of FFAs in therapeutic protein formulations can lead to the formation of undesirable visible and subvisible particles under drug product storage conditions (5, 18–20). The impact of polysorbate-derived FFA particles on therapeutic protein quality and immunogenicity remains to be clarified; however, there have been ongoing efforts to characterize polysorbate degradation under various pharmaceutically relevant conditions and potential impact on the quality and safety of the products they stabilize (21, 22). The exact relationship between fatty acid composition in polysorbate, degradation of polysorbates to release FFAs in formulations, impact of pH on polysorbate degradation, formation of fatty acid particles, and their impact on protein quality remains undefined due to limited studies and available data. In our previous study, we investigated the fatty acid composition in PS80, degradation of PS80 under enzyme-induced stress conditions, and potential impact on therapeutic proteins (19). In this study, we compared the hydrolytic degradation of PS20 NF showing heterogeneity in fatty acid composition and polysorbate 20 with high % lauric acid (PS20 PLA) using two hydrolytic enzymes (esterase and lipase) in two therapeutic protein formulations at different pH values and temperatures. Formulations were prepared with and without therapeutic proteins, subjected to various stress conditions, and analyzed for FFAs release, subvisible particle formation, protein integrity, and biological activity.

## Materials and Methods

PS20 NF was purchased from Spectrum Biochemical. Non-compendial, non-commercial research grade polysorbate 20 with high % lauric acid (PS20 PLA) was kindly provided by Croda Inc. The PS20 PLA from Croda used in our study contains mostly lauric acid (C12) in its fatty acid composition with reported value of 99.6% lauric acid whereas the PS20 NF lot used in our study was reported to have 52.2% lauric acid. Bevacizumab and trastuzumab emtansine (trastuzumab-DM1, or T-DM1) were purchased from McKesson Corporation. Porcine liver esterase (product # E2884-5KU) and lipoprotein lipase from Burkholderia (Product # L19656-15 mg) were purchased from Sigma‒Aldrich. Target cells, effector cells, and critical reagents for the ADCC assay were purchased from Promega. All other chemicals were purchased from Sigma‒Aldrich unless otherwise specified.

### Measurement of free fatty acids (FFAs)

Two biologics (bevacizumab and T-DM1) that contain PS20 in the marketed drug formulation were selected in this study to determine the impact of PS20 degradation on protein quality. Bevacizumab formulation buffer (60 g/L trehalose dihydrate, 5.8 g/L sodium phosphate monobasic monohydrate, 1.2 g/L sodium phosphate dibasic anhydrous, 0.04% w/v polysorbate 20, pH 6.2) and T-DM1 formulation buffer (1.62 g/L sodium succinate, 60 g/L sucrose, 0.02% w/v polysorbate 20 pH 5.0) were prepared with PS20 NF and PS20 PLA without therapeutic drugs. The above formulations were also prepared with 1 mg/mL bevacizumab and T-DM1 to assess the impact of PS20 degradation on protein stability and biological activity. To reformulate biologic drugs, preformulated bevacizumab and T-DM1 were sufficiently diluted in the freshly prepared corresponding formulation buffers without PS20 to maintain the PS20 concentration below its CMC value of 0.06–0.07% and spin dialyzed. In the final two rounds of spin dialysis, each drug was diluted and dialyzed against the corresponding formulation buffer containing either PS20 NF or PS20 PLA. All buffers were filtered through 0.22 µm filters after preparation.

Degradation of PS20 was confirmed by enzymatic hydrolysis of PS20 and measurement of released FFA concentration in formulation buffers. For the enzymatic hydrolysis of PS20, formulation buffers with and without 1 mg/mL of the corresponding drugs were spiked with 1 U/mL esterase or 250 U/mL lipase and incubated at 4°C and 37°C (19). The FFA concentration was measured using the enzymatic colorimetric Non-Esterified Fatty Acid assay (NEFA) kit from Wako Diagnostics according to the manufacturer’s instructions using a SpectraMax i3 microplate reader (Molecular Devices). This method relies on the acylation of coenzyme A (CoA) and oxidation of CoA by acyl-CoA oxidase to produce hydrogen peroxide. The added peroxidase allows for the oxidative condensation of 3-methyl-N-ethyl-N-(β-hydroxyethyl)-amine with 4-aminoantipyrine to form a purple-colored end product that can be measured colorimetricaly at 550 nm. The assay has a broad linearity range from 0.01–4.00 mEq/L.

### Particle analysis

Subvisible particles in formulation buffer after enzymatic degradation of PS20 were analyzed using a microfluidic imaging (MFI) 5200 Flow system equipped with a 100 µm flow cell (ProteinSimple, San Jose, CA). Data were acquired and processed with MFI view software (MVSS). Samples were measured in triplicate with or without dilution. The sample purge volume was set at 0.20 mL, and 0.51 mL was analyzed with a flow rate of 0.10 mL/min. Particles ranging from 1 to 100 µM size were reported as the number of particles/mL.

### Antibody Dependent Cellular Activity (ADCC) Assay

The ADCC activity of bevacizumab and T-DM1 was measured using the Promega VEGF bioassay kit (GA2001 and GA2005) and ADCC Reporter Bioassay kit (G7010 and G7018) following the manufacturer’s instructions.

### T-DM1 ADCC activity

Target cells, HER2 + SKBR3 cells, were maintained and proliferated in McCoy’s 5a medium supplemented with 10% FBS under a humidified atmosphere with 5% CO_2_ at 37°C. Target cells were harvested by centrifugation at 100 X g for 10 min and resuspended in ADCC assay buffer (RPMI 1640 supplemented with 2 mM L-glutamine and 1% low-IgG FBS) at a concentration of 300,000 cells/mL, and 100 µL of resuspended cells was seeded in each well of a 96-well plate at a cell concentration of 30,000 cells/well. Plates were incubated in a cell culture incubator for 6–8 h before adding antibody and effector cells. A series of threefold dilutions of T-DM1 was prepared in a 96-well plate starting with 3 µg/mL in ADCC assay buffer. Using a multichannel pipette, 95 µL of culture medium was removed from each well of the assay plate, and 30 µL of serially diluted antibody solution was added to each well of the assay plate containing effector cells.

Effector cells were maintained in RPMI 1640 containing 10% FBS, 100 µg/mL hygromycin, 250 µg/mL G-418 sulfate solution, 1 mM sodium pyruvate and 0.1 mM MEM nonessential amino acids. Cells were harvested by centrifugation at 100 X g for 10 min and resuspended in ADCC assay buffer at a concentration of 6X10^6^ cells/mL. Twenty-five microliters of effector cells were added to each well of the assay plate with a target cell to effector cell ratio of 1:5 (target cells:effector cells = 30,000:150,000) and incubated overnight from 16–18 h in a 37°C CO_2_ tissue culture incubator. Assay plates were removed from the incubator, and 75 µL of prewarmed Bio-Glo Luciferase assay reagent at room temperature was added to each well. Assay plates were incubated at room temperature for 10–20 min, and luminescence was measured in a well-scan mode with 0.5 s integration time using a SpectraMax i3 microplate reader (Molecular Devices). EC50 values were determined using GraphPad Prism curve fitting software.

### Bevacizumab ADCC activity (VEGF Bioassay)

The KDR/NEFT-RE HEK293 cells were maintained in DMEM containing 10% FBS and 50 µg/mL hygromycin B. Cells were harvested and resuspended in prewarmed assay buffer to a concentration of 3 × 10^6^ cells/mL. Using a multichannel pipette, 25 µL of cell suspension was added to each of the inner 60 wells in a 96-well plate. A serial threefold dilution of bevacizumab was prepared for each sample with a starting concentration of 18 µg/mL. Using a multichannel pipette, 25 µL of antibody dilution solution and 25 µL of recombinant VEGF protein with a concentration of 3 × its EC_80_ value (EC_80_ = 20 ng/mL) were dispensed to each designated well in the assay plate. The assay plates were incubated in a CO_2_ tissue culture incubator at 37°C for 6 h. After incubation, 75 µL of prewarmed Bio-Glo Luciferase assay reagent at room temperature was added to each well. Assay plates were incubated at room temperature for 10–20 min, and luminescence was measured in a well-scan mode with 0.5 s integration time. EC50 values were determined using GraphPad Prism curve fitting software.

### Size exclusion ultra-performance liquid chromatography (SE-UPLC)

Size variants in bevacizumab and T-DM1 before and after treatment with enzymes were determined by size exclusion chromatography (SEC) using a Waters Acquity UPLC system (Milford, MA). SEC was performed using an Acquity UPLC Protein BEH SEC 200 Å Column (4.6 × 150 mm, 1.7 µm) and 50 mM sodium phosphate, 150 mM NaCl, pH 6.8 as a mobile phase. Protein elution was monitored with UV absorption at 220 nm and 280 nm using Waters Acquity PDA detector.

### Circular dichroism (CD) spectroscopy

Bevacizumab and T-DM1 samples at 1 mg/mL in their respective buffer containing either PS20 NF or PS20 PLA with and without esterase and lipase enzymes were incubated at 37°C for 4 weeks. Samples were then buffer exchanged to phosphate buffer at pH 7.5, adjusted to a concentration of 1 mg/mL and analyzed by CD spectroscopy in 0.5 mm path-length quartz cuvettes using JASCO-1700 equipped with a EXOS liquid cooling system and temperature-controlled holder. Data acquisition was performed using range of 260–185 nm at 20°C, and 40—80°C every 5°C.

### Statistical analysis

Data were analyzed by GraphPad Prism 6. Statistical significance was determined using one-way analysis of variance (ANOVA) with Tukey’s multiple comparison tests. A *p* value of < 0.05 was considered statistically significant.

## Results

### Effect of therapeutic proteins in formulations for enzymatic hydrolysis of PS20 and formation of subvisible particles

**Fig. 1:**
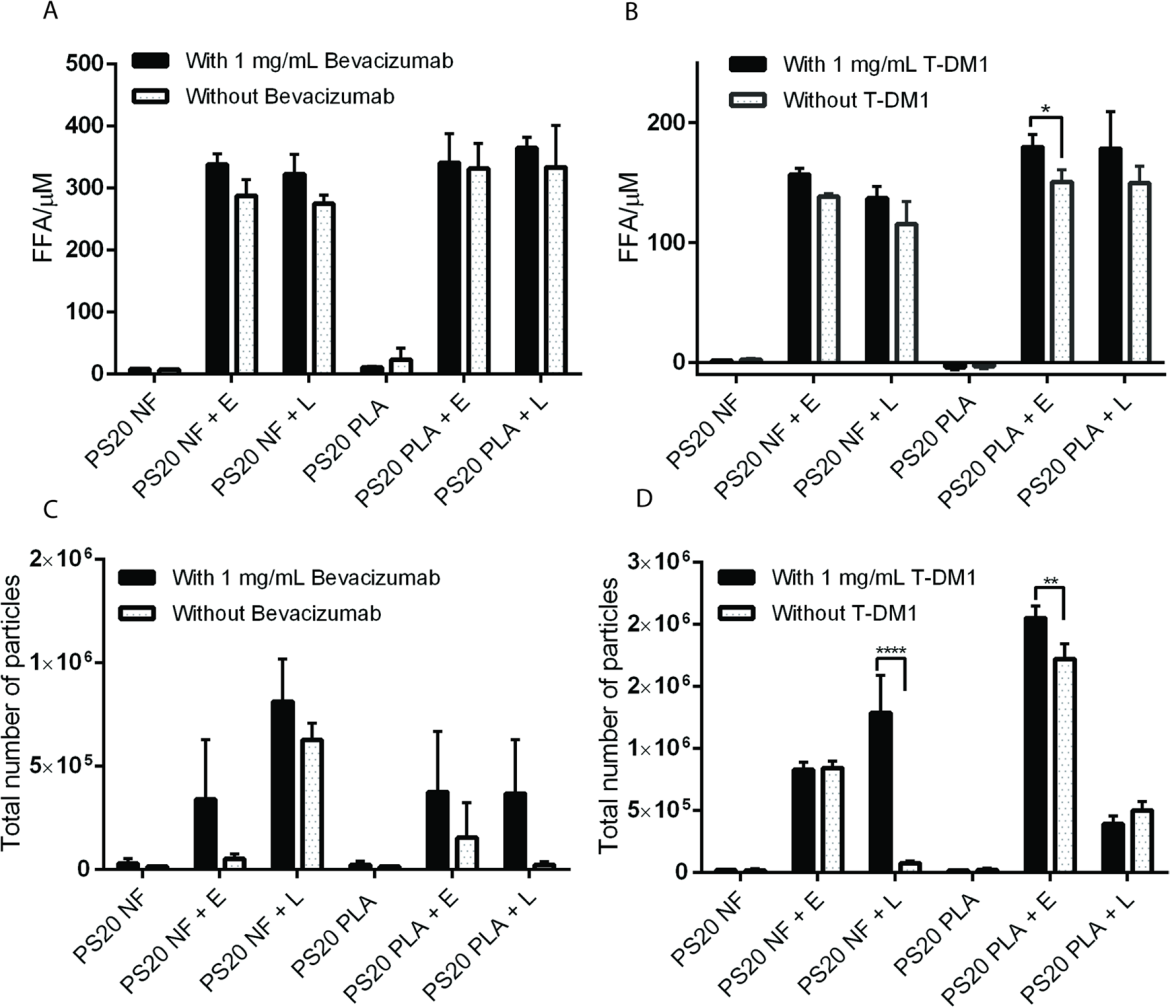
Release of FFAs and formation of subvisible particles in PS20-containing formulations at 4°C. Formulations were prepared with and without 1 mg/mL bevacizumab or T-DM1 and treated with 1 U/mL esterase or 250 U/mL lipase at 4°C for 4 weeks. FFAs and subvisible particles were determined for bevacizumab (panels A and C) and T-DM1 (panels B and D). Statistical analysis was performed for each enzyme-treated sample with and without therapeutic proteins to determine the impact of therapeutic proteins on the release of FFAs from PS20 and the formation of FFA particles. The total particles or size specific particles on Y-axis represent the number of particles/mL. E = esterase, L = lipase (**** p < 0.0001, ** p < 0.01, *p < 0.05)

We prepared formulations of bevacizumab and T-DM1 with and without bevacizumab and T-DM1 and treated them with either 1 U/mL esterase or 250 U/mL lipase at 4°C to compare the release of FFAs and the formation of subvisible particles in each formulation with and without therapeutic proteins. Esterase (1 U/mL) and lipase (250 U/mL) concentrations were selected based on the optimal ability to release the maximum amount of FFAs from PS20 (Supplementary Fig. S1). While 250 U/mL lipase was required for the maximum release of FFA from PS20, use of 0.5U/mL, 1U/mL or 2U/mL did not show significant difference in the release of FFA. Consistent with our previous publication on polysorbate 80 and other reports, we selected 1U/mL esterase in our study (18, 19). We observed comparable levels of FFA in each formulation after each enzyme treatment, and the presence of therapeutic proteins in the formulations did not significantly impact the release of FFAs from PS20 (Fig. 1A and B). A statistically significant increase in total FFA concentration was observed only for esterase-treated PS20 PLA containing the T-DM1 formulation in the presence of therapeutic proteins compared to the formulation without T-DM1. Slight variations in the total number of subvisible particles with and without therapeutic proteins were observed, but only the lipase-treated PS20 NF-containing formulation and esterase-treated PS20 PLA-containing T-DM1 formulation showed statistically significant differences (Fig. 1C and D).

### Effect of fatty acid composition in PS20 on the release of FFAs and formation of subvisible particles

**Fig. 2:**
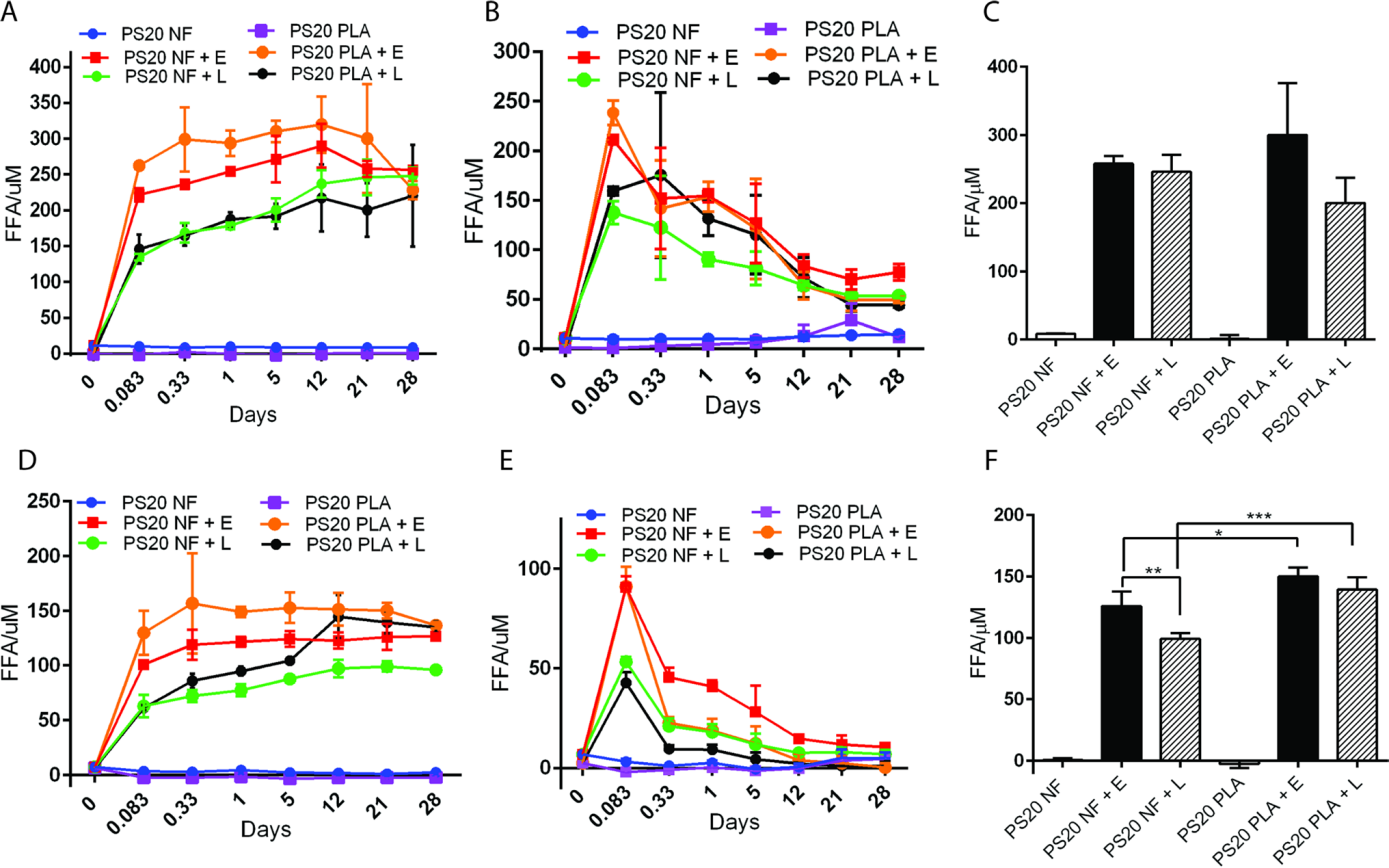
Release of FFAs from PS20 depends on the fatty acid composition and hydrolytic enzymes. Bevacizumab and T-DM1 formulations containing PS20 NF or PS20 PLA were prepared without therapeutic proteins, treated with 1 U/mL esterase or 250 U/mL lipase, and incubated at 4°C and 37°C. The release of FFA was monitored for 4 weeks at 4°C and 37°C for bevacizumab (A and B) and T-DM1 (D and E). Panels C and F represent FFA levels for bevacizumab and T-DM1, respectively, at 4°C from panels A and D after 4 weeks of enzyme treatment. Statistical analysis was performed to compare two enzymes for the release of FFAs from PS20 NF or PS20 PLA and to compare PS20 NF and PS20 PLA for the release of FFAs by each enzyme. E = esterase, L = lipase (*** p < 0.001, ** p < 0.01, *p < 0.05)

Because we observed comparable FFA concentrations with and without therapeutic proteins in formulations after treatment with hydrolytic enzymes, we carried out most of our experiments without using therapeutic proteins in the formulation buffers to perform advanced characterization of the enzymatic hydrolysis of PS20, the release of FFAs in the formulation, and the formation of subvisible particles under relevant conditions. The trend on FFA and subvisible particles over time were comparable at room temperature or 37°C; therefore, to be consistent with our previous studies on therapeutic protein stability in PS80 containing formulation, we selected 37°C as a physiologically relevant temperature with implications on in-use stability during infusions instead of room temperature (19). Treatment of PS20 NF or PS20 PLA containing bevacizumab and T-DM1 formulations with 1 U/mL esterase or 250 U/mL lipase produced significantly greater levels of FFA compared to the untreated control (Fig. 2). The enzyme-treated bevacizumab formulation showed a greater increase in FFA levels than the T-DM1 formulation, which could be due to the presence of a higher concentration of PS20 in the bevacizumab formulation (0.04% w/v) than in the T-DM1 formulation (0.02% w/v). There was a steady increase in FFA concentrations for lipase-treated formulations at 4°C. However, for esterase-treated formulations, maximum FFA concentrations were achieved within 8 h, remained constant for up to three weeks, and then started to decrease (Fig. 2A and D). Generally, each enzyme treatment produced different levels of FFA from PS20 NF and PS20 PLA, with higher levels of FFAs detected in PS20 PLA-containing formulations compared to PS20 NF-containing formulations. However, the lipase-treated bevacizumab formulation was an exception, where PS20 NF showed higher levels of FFA after 2 weeks of incubation. The differences in FFA levels were not statistically significant between PS20 NF- and PS20 PLA-containing bevacizumab formulations after treatment with esterase or lipase for 4 weeks at 4°C, but these results were significantly different for T-DM1 formulations (Fig. 2C and F). At 37°C, after an initial increase, FFA levels continued to decrease for up to 3 weeks and then remained steady up to 4 weeks in all formulations regardless of the grades of PS20 and enzymes used in our study (Fig. 2B and E). We did not measure FFA levels beyond 28 days in our experiments.

**Fig. 3:**
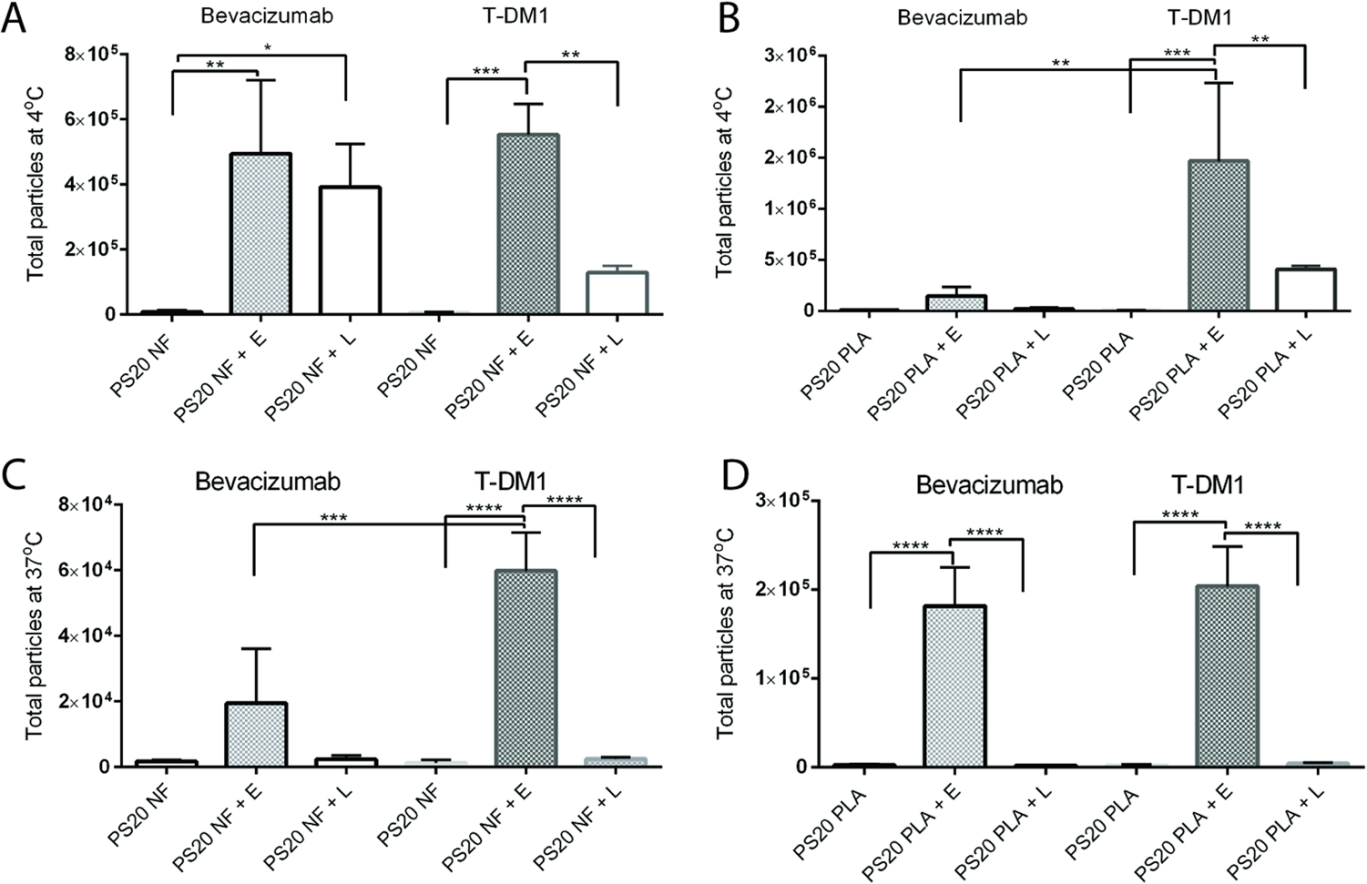
Comparison of PS20 NF and PS20 PLA degradation to form subvisible particles in different formulations at 4°C or 37°C in the presence of esterase or lipase. The total number of subvisible particles was compared between bevacizumab and T-DM1 formulations in panels A-D. For statistical analysis, the results from enzyme-treated samples were compared with the control for each drug. The results were also compared between two enzymes for each drug and for the same enzyme between two drugs. The total particles or size specific particles on Y-axis represent the number of particles/mL. E = esterase, L = lipase (**** p < 0.0001, *** p < 0.001, ** p < 0.01, *p < 0.05)

Following FFA analysis, we measured subvisible particles in the same formulations using MFI. We compared subvisible particles between bevacizumab and T-DM1 formulations for each enzyme treatment at 4°C and 37°C. Esterase or lipase treatment produced a significantly greater number of subvisible particles in all samples compared to untreated controls, and a greater number of subvisible particles were observed at 4°C compared to 37°C for all formulations (Fig. 3A-D, Tables S1 and S2). The total number of subvisible particles for the esterase-treated T-DM1 formulation was significantly greater than that for the esterase-treated bevacizumab formulation for PS20 PLA at 4°C and PS20 NF at 37°C (Fig. 3B and C). Esterase treatment selectively produced a significantly greater number of subvisible particles than lipase treatment in all T-DM1 formulations at both 4°C and 37°C, but in the bevacizumab formulation, we observed a significant difference in the total number of subvisible particles between esterase and lipase treatment only with the PS20 PLA-containing formulation at 37°C (Fig. 3A-D, Tables S1 and S2).

**Fig. 4:**
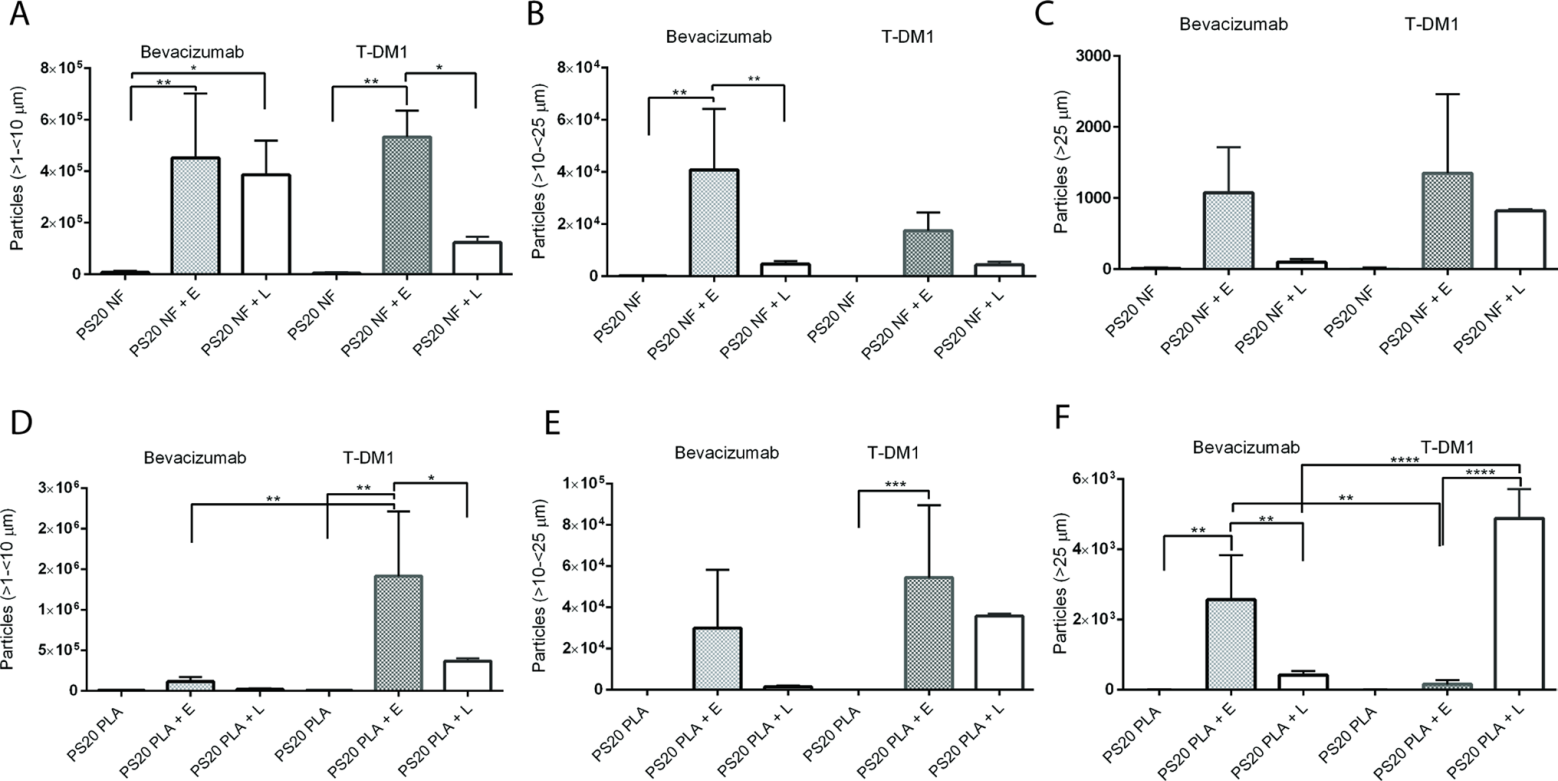
Comparison of PS20 NF and PS20 PLA degradation to form subvisible particles in different formulations at 4°C or 37°C in the presence of esterase or lipase. The total number of subvisible particles from Figure panels 3A-3D were further analyzed by sizes of > 1- < 10 µm, > 10- < 25 µm and > 25 µm. For statistical analysis, the results from enzyme-treated samples were compared with the control for each drug. The results were also compared between two enzymes for each drug and for the same enzyme between two drugs. The total particles or size specific particles on Y-axis represent the number of particles/mL. E = esterase, L = lipase (**** p < 0.0001, *** p < 0.001, ** p < 0.01, *p < 0.05)

We observed a greater number of subvisible particles at 4°C compared to 37°C; therefore, we performed further size-based analysis of subvisible particles at 4°C (Fig. 4A-F). Particle sizes of > 1—< 10 µm showed a similar pattern to the total number of particles (Fig. 4A and E). There were no significant differences in total > 10- < 25 µm particles and > 25 µm particles for PS20 NF-containing bevacizumab and T-DM1 formulations after esterase or lipase treatment; however, for PS20 PLA-containing formulations, there were significant differences in the number of > 25 µm particles between the two enzyme treatments within each formulation or between the two formulations (Fig. 4C and F). Lipase treatment produced a significantly greater number of > 25 µm particles than esterase treatment in the PS20 PLA containing the T-DM1 formulation.

### pH-dependent release of free fatty acids and formation of particles

**Fig. 5:**
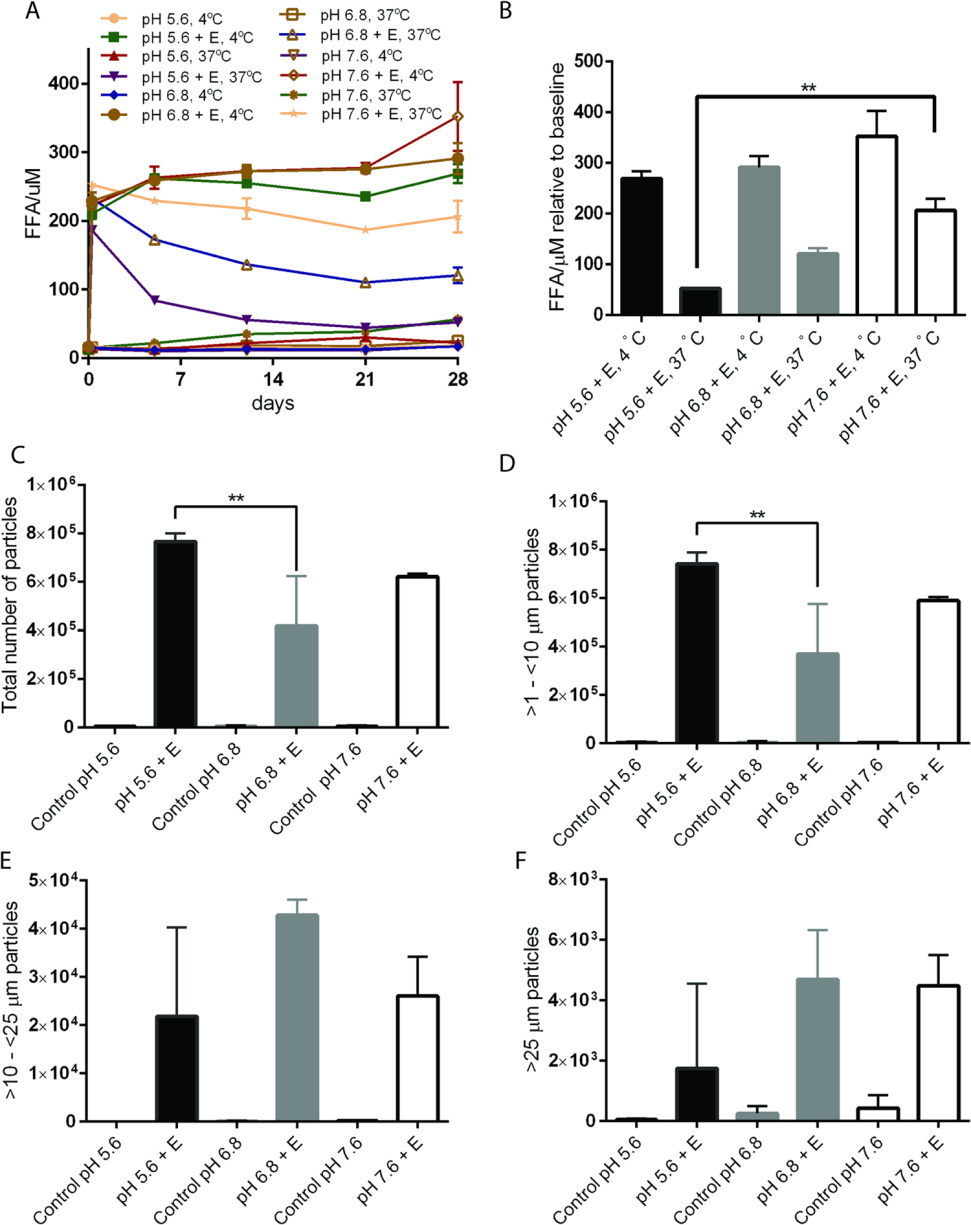
pH-dependent release of FFAs and formation of subvisible particles. Formulation buffers containing the same excipients with 0.02% PS20 NF at pH 5.6, 6.8 and 7.6 were prepared and treated with 1 U/mL esterase at 4°C and 37°C. The release of FFAs was monitored for 4 weeks (A) under both storage conditions. The FFA concentration (B) and total number of particles (C) were determined after 4 weeks. Subvisible particles were determined using MFI and analyzed for total number of particles (C), > 1- < 10 µm (D), > 10- < 25 µm (E), and > 25 µm particles (F) expressed as number of particles/mL. Statistical analysis was performed within esterase-treated samples at 37°C or at 4°C at different pH values. E = esterase, L = lipase (** p < 0.01, *p < 0.05)

The bevacizumab formulation prepared at pH 6.2 contained a higher concentration of PS20 (0.04% w/v) than the T-DM1 formulation (0.02% w/v) prepared at pH 5.0. Based on the PS20 composition and FFA release after esterase or lipase hydrolysis, we hypothesized that the bevacizumab formulation would produce more FFA particles than the T-DM-1 formulation, but the results did not support this hypothesis. We determined comparable levels of the total number of subvisible particles between bevacizumab and T-DM1 formulations containing PS20 NF with esterase treatment and a greater number of subvisible particles in PS20 PLA containing T-DM1 formulations compared to the bevacizumab formulation (Fig. 3A-D). This finding indicates that either pH or the composition of excipients in the formulation buffer may be driving the formation of FFA particles. Marketed biologics are generally formulated in buffer with pH ranging from 4.0–7.4 and majority of drug formulations have pH in the range of 5 and 6. Therefore, to address potential impact of pH on PS20 degradation in biotherapeutic drugs formulations, we tested three pharmaceutically relevant pH for our study. To evaluate whether pH is a critical factor for the formation of FFA particles, we prepared three formulations at pH 5.6, 6.8, and 7.4 with the same formulation excipients and 0.02% (w/v) PS20. After treatment with esterase, we observed an increase in the levels of FFA with increasing pH (more FFAs at pH 7.4 and 6.8 than at pH 5.6) at both 4°C and 37°C (Fig. 5A and B). However, a greater number of total and 1 to 10 µm subvisible particles were observed at pH 5.6 than at pH 6.8 or 7.6 (Fig. 5C and D). At a near neutral pH of 6.8, a greater number of ≥ 10 µm subvisible particles were observed compared to those at pH 5.6 (Fig. 5E and F). The total number of particles was significantly greater at 4°C than at 37°C in all three formulations (Supplementary Fig. S2A). Consistent with the results at 4°C, we observed a significantly greater number of subvisible particles at pH 5.6 than at pH 6.8 or pH 7.6 at 37°C (Supplementary Fig. S2B). These data indicate that the hydrolysis of PS20 and the formation of subvisible particles are influenced by the temperature and pH of the drug formulations.

### Effect of dilution and temperature on particle formation

**Fig. 6:**
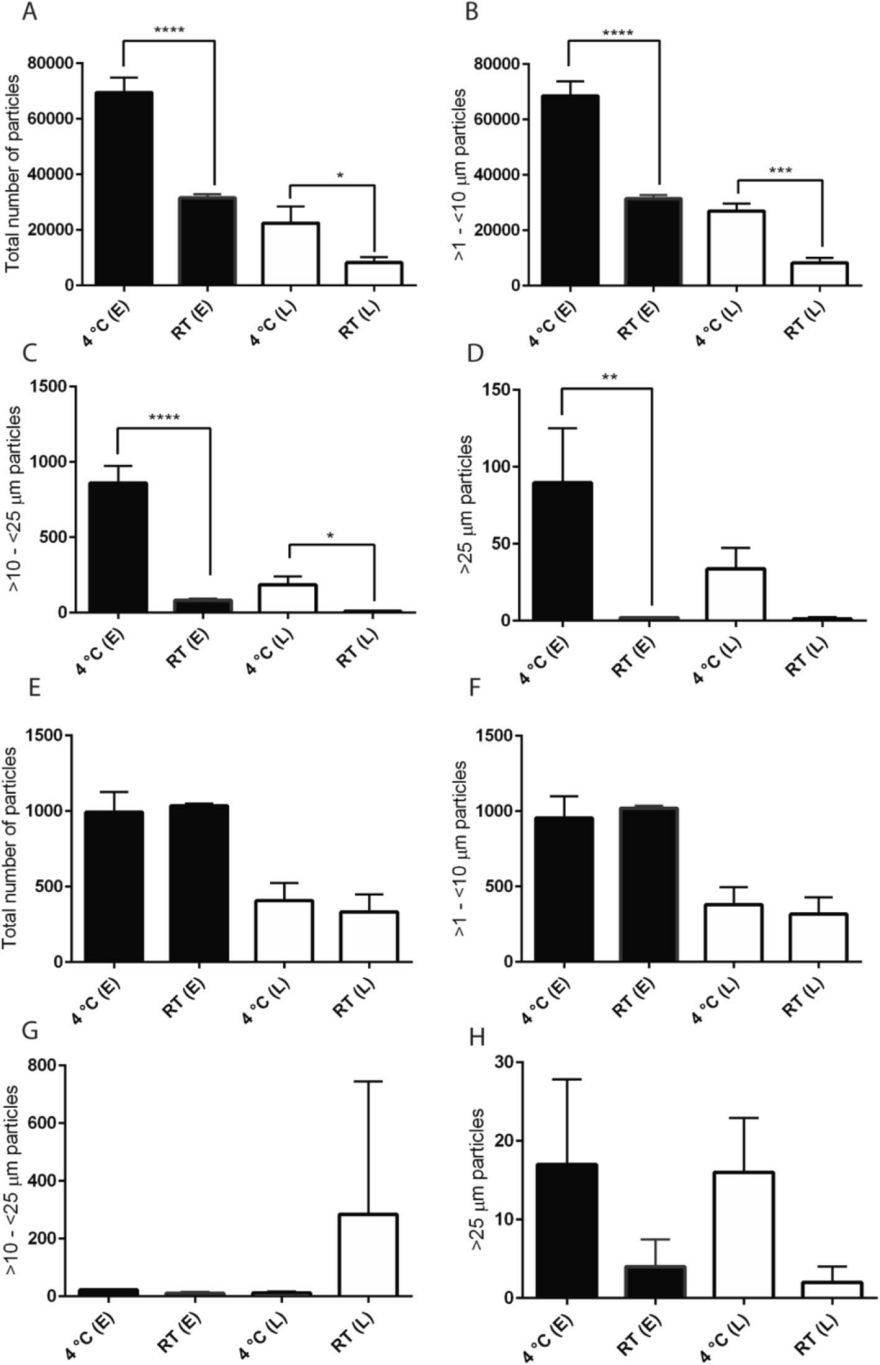
Effect of dilution and storage temperature on FFA particles. Bevacizumab formulations without therapeutic protein were treated with 1 U/mL esterase and 250 U/mL lipase for 21 days at 4°C and diluted 10X with saline. Diluted samples were incubated either at 4°C or at room temperature for 2 h before analyzing subvisible particles using MFI. Panels A-D represent the total number, > 1- < 10 µm, > 10- < 25 µm, and > 25 µm subvisible particles, respectively, for bevacizumab formulation containing PS20 NF. Panels E–H represent the total number, > 1- < 10 µm, > 10- < 25 µm, and > 25 µm subvisible particles expressed as number of particles/mL, respectively, for bevacizumab formulation containing PS20 PLA. E = esterase, L = lipase (**** p < 0.0001, *** p < 0.001, ** p < 0.01, *p < 0.05)

Most biotherapeutic drugs are diluted in a compatible diluent at ambient temperature and administered to the patients within a few hours of dilution, as specified in the package insert based on established supporting in-use stability data for specific drugs. To determine whether dilution and storage at room temperature has any impact on FFA and subvisible particle formation, we prepared bevacizumab formulations with 0.04% (w/v) PS20 NF or PS20 PLA and treated them with esterase and lipase at 4°C for 4 weeks. An aliquot was taken from each sample and diluted 10X with saline stored at 4°C or room temperature. Diluted formulations were stored at 4°C or room temperature for 2 h before particle analysis using MFI. We observed a significant reduction in the total number of subvisible particles for esterase- or lipase-treated PS20 NF-containing formulations when stored at RT compared to the samples stored at 4°C (Fig. 6A-D). For PS20 PLA-containing formulations, we did not observe such a reduction for total or 1 to 10 µm particles; however, > 25 µm particles were reduced when stored at RT after dilution in saline (Fig. 6E-H). These results indicate that FFA particles from different grades of PS20 degradation behave differently when diluted with saline at room temperature and that PS20 NF exhibited lower particle formation under the conditions tested.

### Effect of polysorbate degradation on the structural integrity of therapeutic proteins

**Fig. 7:**
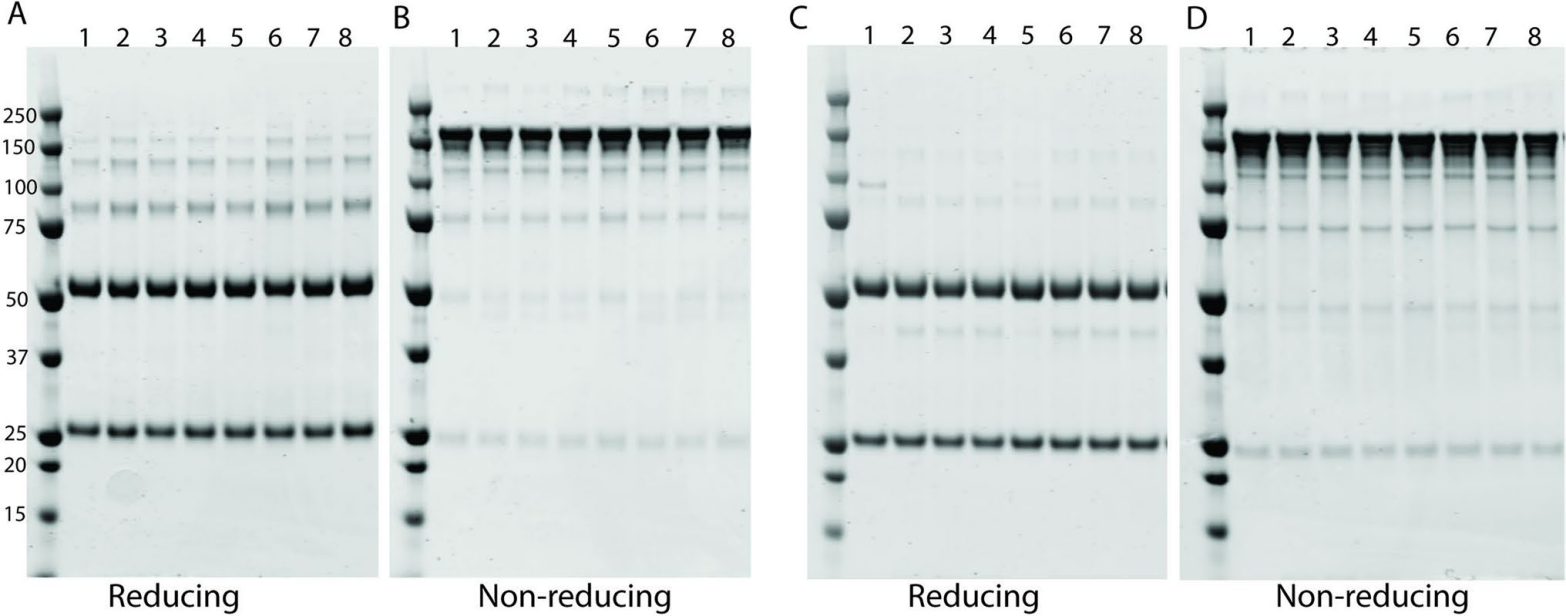
Effect of polysorbate degradation on the stability of therapeutic proteins against aggregation or degradation. All formulations were prepared with therapeutic proteins. Samples prepared after treatment with esterase or lipase enzymes at 37°C for 4 weeks were analyzed by SDS‒PAGE for T-DM1 (A and B) and bevacizumab (C and D) under reduced and nonreduced conditions. Lanes 1–4 represent PS20 NF-containing formulations for both T-DM1 and bevacizumab; Lane 1: control at 4°C, Lane 2: control at 37°C, Lane 3: esterase-treated sample at 37°C, Lane 4: lipase-treated sample at 37°C. Lanes 5–8 represent PS20 PLA-containing formulations for both T-DM1 and bevacizumab; Lane 5: control at 4°C, Lane 6: control at 37°C, Lane 7: esterase-treated sample at 37°C, Lane 8: lipase-treated sample at 37°C

To determine the impact of polysorbate degradation on protein integrity, we ran SDS‒PAGE under reducing and nonreducing conditions for bevacizumab and T-DM1 samples with and without esterase or lipase treatment for 4 weeks at 37°C. We did not observe differences in protein bands before and after treatment with esterase or lipase (Fig. 7A-D). We also did not observe any difference between control samples stored at 4°C (lane 1 for each gel) and samples stored at 37°C. Similarly, we did not observe any band patterns suggestive of aggregated or degraded products for bevacizumab and T-DM1 in SDS‒PAGE after treatment with esterase and lipase for more than 6 months at 4°C (data not shown). It is noted that SDS-PAGE analysis is limited to detection of only covalently linked protein aggregates and protein aggregates formed by the non-covalent interaction will not be detected in the SDS-PAGE analysis.

**Fig. 8:**
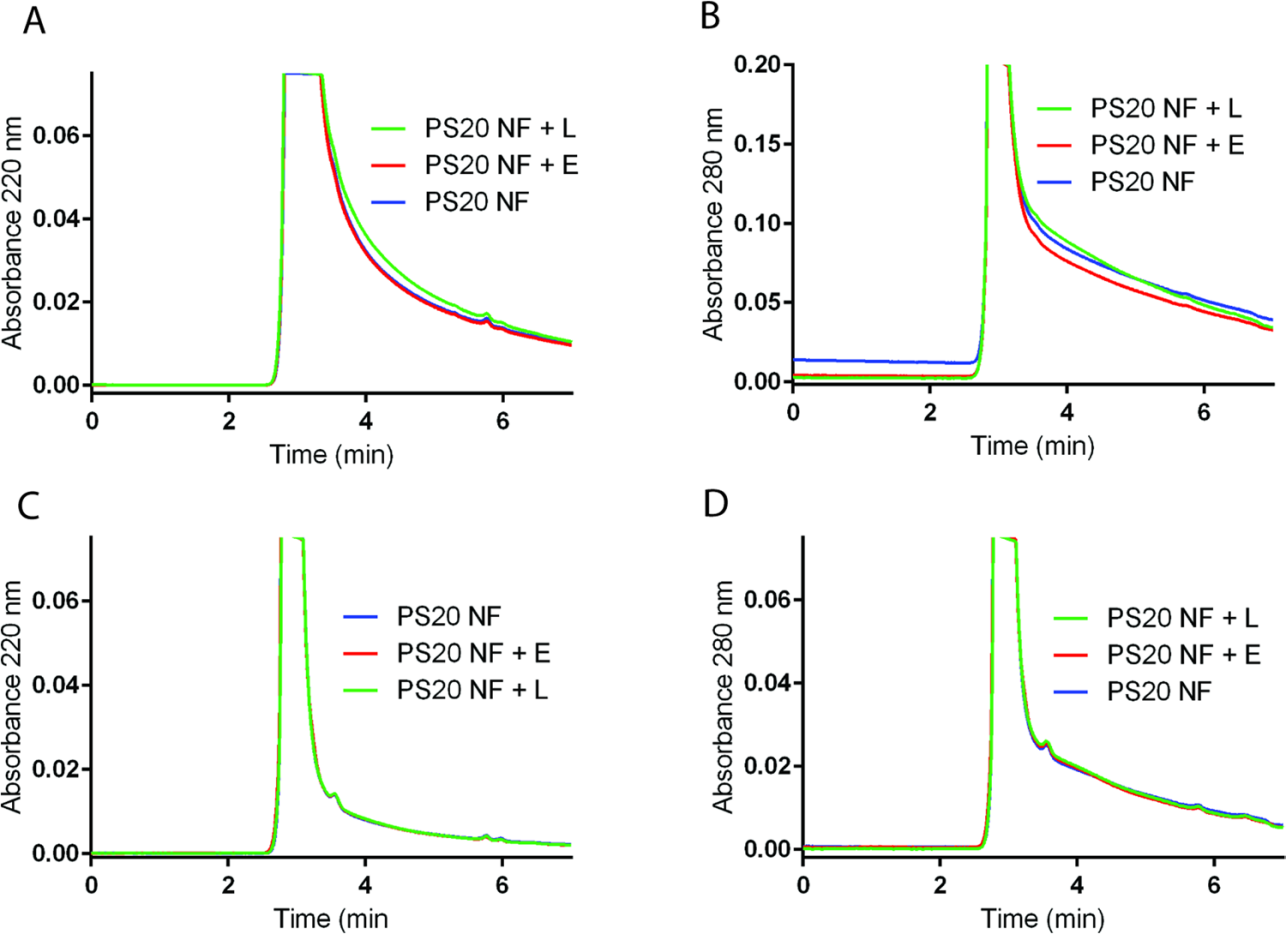
Effect of PS20 degradation on the stability of therapeutic proteins and the formation of HMWs. Formulations prepared with therapeutic proteins were treated with 1 U/mL esterase or 250 U/mL lipase enzymes at 37°C for 4 weeks and analyzed by SEC-UPLC. Representative Zoomed view of SEC-UPLC chromatograms for T-DM1 (A and B) and bevacizumab (C and D) with UV detection at 220 nm and 280 nm. Chromatograms were normalized and overlaid for side-by-side comparison. E = esterase, L = Lipase

We further analyzed stress stability samples using SEC-UPLC to determine the potential formation of high molecular weight species (HMWs) of therapeutic proteins caused by PS20 degradation in formulations. We did not observe any differences in SEC-UPLC chromatograms before and after enzyme-induced degradation of PS20 at 37°C (Fig. 8A-D, Fig S4). There were no additional peaks for HMWS or degradation products for either bevacizumab or T-DM1 samples after treatment with esterase or lipase for 4 weeks at 37°C.

### Impact of polysorbate degradation on secondary structures of proteins

**Fig. 9:**
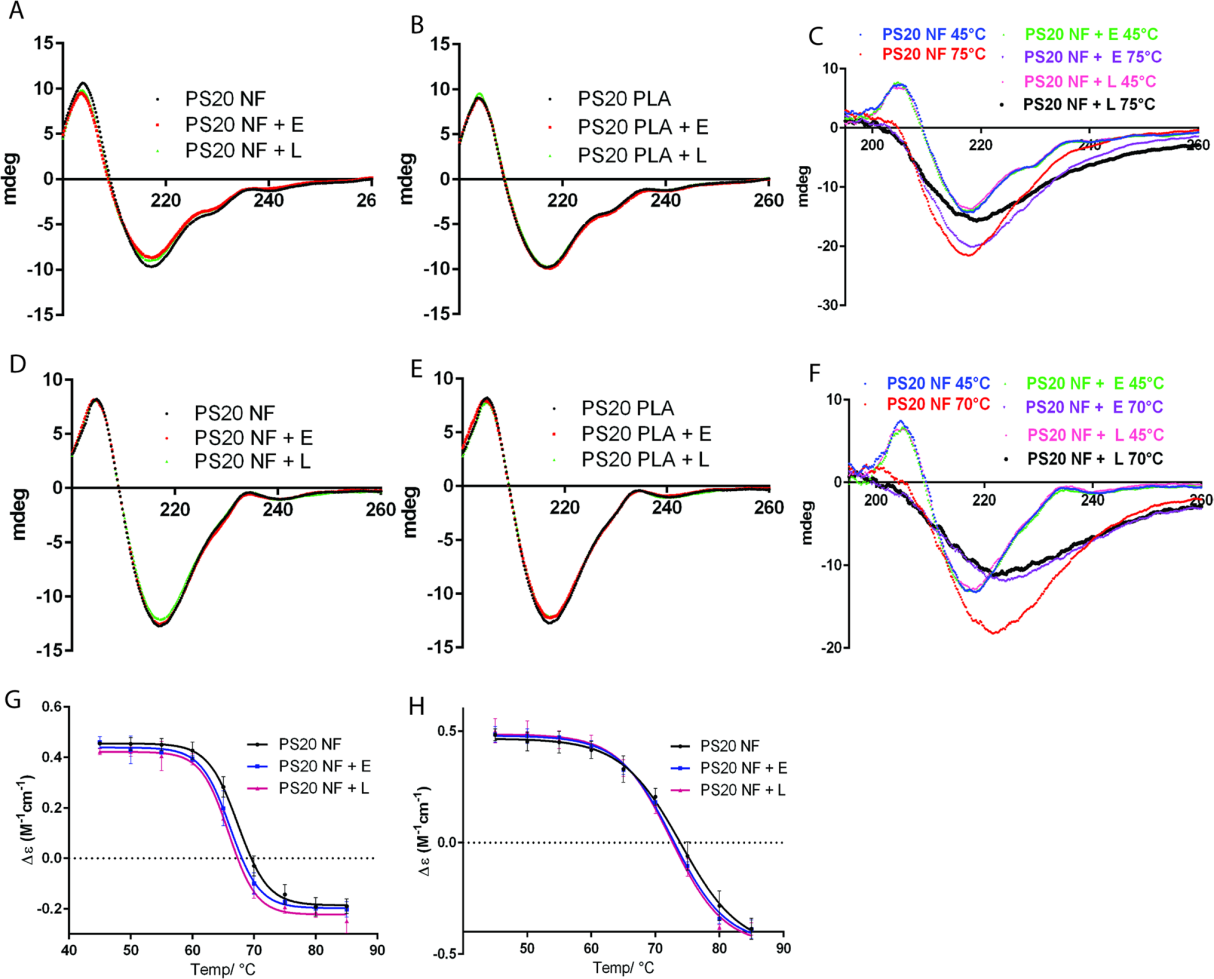
Effect of polysorbate degradation on the secondary structures of therapeutic proteins. Samples were treated with 1 U/mL esterase or 250 U/mL lipase and incubated for 4 weeks at 37°C before analysis. Representative CD spectra of T-DM1 (1 mg/mL) in PS20 NF-containing formulation (A) and PS20 PLA-containing formulation (B). Representative CD spectra of bevacizumab (1 mg/mL) in the PS20 NF-containing formulation (D) and PS20 PLA-containing formulation (E). CD spectra were collected at 20°C. Samples were also analyzed as a function of temperature from 40°C to 85°C at an interval of 5°C to determine the impact of PS20 degradation on thermal unfolding. Representative CD spectra for T-DM1 at 45°C and 75°C (C) and for bevacizumab at 45°C and 70°C (F) are shown. Molar circular dichroism (Δε) as a function of temperature at 204.6 nm for T-DM1 (G) and at 205.4 nm for bevacizumab (H) were plotted to determine changes in protein folding. E = esterase, L = Lipase

The potential impact of polysorbate degradation on the secondary structure of bevacizumab and T-DM1 was evaluated by far-UV CD spectroscopy recorded at 185–260 nm at 20°C. We did not observe a significant impact on the CD spectra of bevacizumab and T-DM1 after treatment with esterase or lipase for 4 weeks at 37°C (Fig. 9A, B, D, and E). We also evaluated the thermal unfolding of bevacizumab and T-DM1 as a function of temperature from 40°C to 85°C (Fig. 9C, F, G and H). We did not observe any changes in the stability CD spectra of bevacizumab and T-DM1 after hydrolytic degradation of either PS20 NF or PS20 PLA at 20°C; therefore, we only evaluated the PS20 NF-containing formulation for thermal unfolding. We observed noticeable changes in melting curves at 70°C for bevacizumab after esterase or lipase treatment, indicating the impact on the thermal stability of the protein due to the degradation of PS20 in the formulation. The trough at 219 nm widened with a redshift to 223 nm, and the amplitude decreased after lipase or esterase treatment compared to the control (Fig. 9F). This suggests that the degree of unfolding increases for enzyme-treated samples, leading to aggregation at 70°C. Similar changes were observed for T-DM1, but the changes were relatively small (Fig. 9C). Based on the melting curve, the T_m_ values for untreated, esterase-treated, and lipase-treated bevacizumab samples were calculated to be 67.38°C, 65.14°C, and 65.89°C, respectively (Fig. 9H). Similarly, for untreated, esterase-treated, and lipase-treated T-DM1 samples, the T_m_ values were calculated to be 73.94°C, 72.72°C, and 72.46°C, respectively (Fig. 9G). Our data indicate that there may be a slight decrease in the thermal stability of bevacizumab and T-DM1 after hydrolysis of PS20; however, results are not conclusive due to small changes in the thermal stability and additional studies are required.

### Effect of PS20 degradation on the biological activity of therapeutic proteins tested

**Fig. 10:**
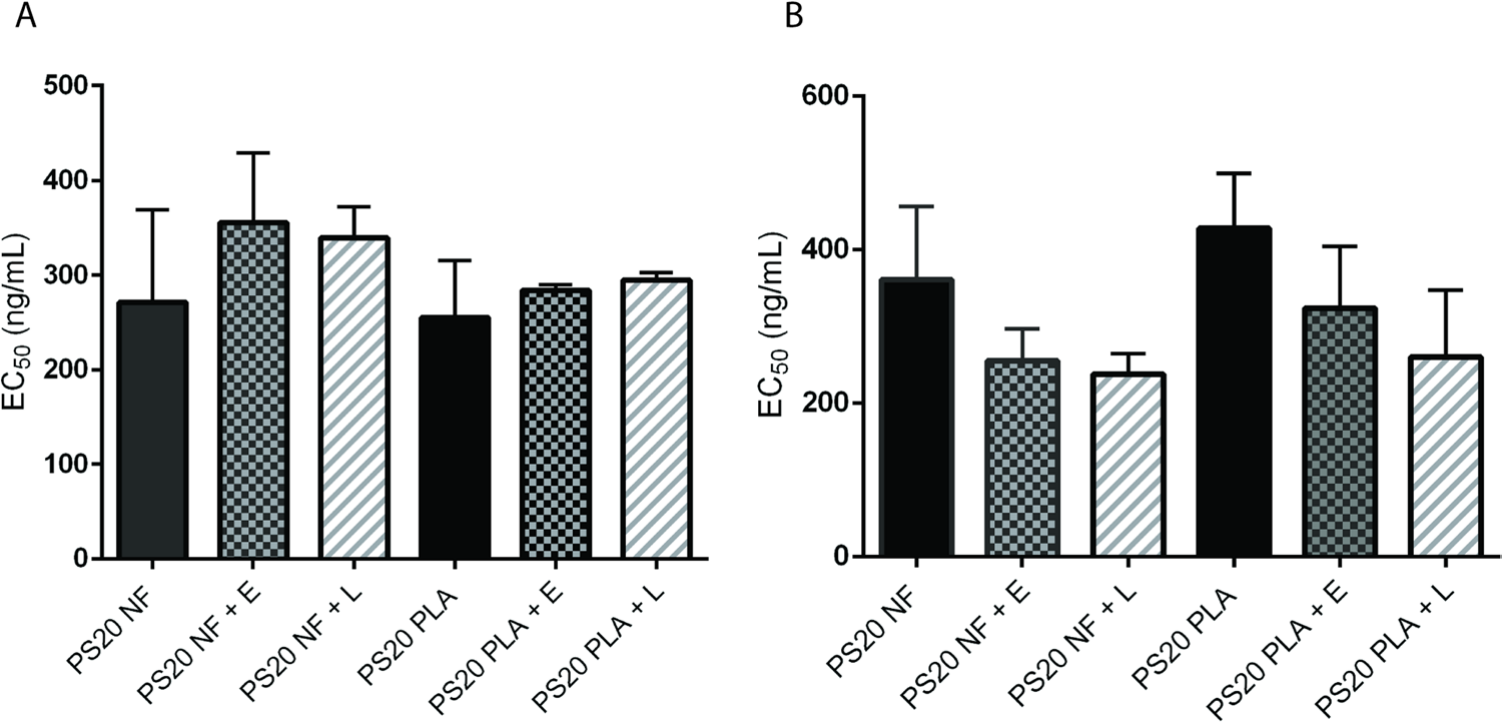
Effect of polysorbate degradation on the biological activity of therapeutic proteins. ADCC activity for T-DM1 (A) and bevacizumab (B) was determined after treatment with 1 U/mL esterase or 250 U/mL lipase for 4 weeks at 37°C. Data shown are mean EC50 values ± SD from n = 3. No significant differences in ADCC activity were observed between the control and enzyme-treated samples. E = esterase, L = Lipase

We observed a greater number of subvisible particles at 4°C under enzyme-induced hydrolysis, but these particles were significantly reduced when samples prepared at 4°C were stored at room temperature or 37°C. Therefore, to evaluate both temperature and hydrolytic enzyme-induced stress stability on protein function, we evaluated the biological activity of PS20 NF or PS20 PLA containing bevacizumab and T-DM1 formulations after esterase and lipase treatment for 4 weeks at 37°C. Hydrolytic degradation of PS20 NF or PS20 PLA at 37°C did not significantly impact the ADCC activity of bevacizumab and T-DM1 (Fig. 10A and B). Our results indicate that the biological activities of bevacizumab and T-DM1 under the experimental conditions tested are not affected even after the degradation of PS20 and the formation of FFA particles. Slight differences observed in the biological activity with and without hydrolytic degradation of PS20 are likely within the method variability.

## Discussion

USP compendial grade polysorbates are used in several commercial drug formulations supporting biotechnology-derived drug products. These surfactants include a heterogeneous mixture of fatty acids in polysorbates with wide acceptance criteria, resulting in lot-to-lot variations in fatty acid composition in compendial PS20 (4, 6). Several factors, including storage conditions, pH, impurities present in raw materials used in PS20 synthesis, formulation excipients, exposure to light, and impurities present in proteins can impact the stability of polysorbates and subsequently their ability to stabilize therapeutic proteins during formulation and storage (23). In this study, we evaluated the hydrolytic degradation of PS20 to release FFAs and the formation of subvisible particles in formulations and the potential impact on therapeutic protein stability and biological activity. Additionally, we investigated multiple formulation aspects related to PS20 to assess their possible impacts on protein quality including PS20 grade (NF or PLA), hydrolytic HCP contaminant (esterase and lipase), drug product (bevacizumab and T-DM1), formulation, pH, and temperature. PS20 PLA contains relatively high-purity lauric acid (99.6%) compared to the variable composition of lauric acid (40–60%) in PS20 NF (6). Previous studies have identified that residual proteins that copurified with therapeutic proteins as host cell proteins (HCPs) can degrade polysorbates in drug formulations during storage at different rates (9, 10, 24). Therefore, we selected enzymatic hydrolysis as a stress condition in our study using two enzymes. The concentration of monoclonal antibodies in the formulation can influence the degradation of polysorbates and the formation of subvisible particles (16). Our data indicate that the presence of relatively low concentration (1 mg/mL) of therapeutic proteins in the formulation does not significantly impact the release of FFAs by enzymatic hydrolysis of PS20 compared to the formulation without therapeutic proteins (Fig. 1). Therefore, we used formulations prepared without therapeutic proteins in our study to evaluate the hydrolytic degradation of PS20 and FFA particle formation under different conditions. Release of FFAs by esterase hydrolysis of fatty acid ester bonds in PS20 was faster than lipase hydrolysis, producing maximum FFAs within 8 h of incubation. However, for lipase hydrolysis, it took up to four weeks to reach the equivalent level of FFA (Fig. 2A and D). The enzymatic activity has been reported to be low at acidic pH for both esterase and lipase, with maximum activity at neutral pH (25). Esterase is a carboxy ester hydrolase that preferentially act on to hydrolyze ester bonds of water-soluble shorter-chain fatty acids. Esterases catalyze hydrolysis of carboxylic acid esters, phosphoric acid mono-, di-, and tri-esters as well as thioesters. Lipoprotein lipase belongs to the family of triglyceride lipases and hydrolyses triglyceride rich lipoproteins to release fatty acids. It hydrolyzes cholesteryl esters, mono-, di-, and tri-acylglycerols, phospholipids, lysophospholipids and ceramids. Lipases act on a much broader substrate range than esterases, including poorly soluble long-chain fatty acids. (26, 27). Lipases or esterases from different species are known to have different activities based on fatty acid composition in polysorbates. Comparison of *Pseudomonas cepacea* lipase (PCL), *Thermomyces lanuginosus* lipase, and lipase B *Candida antarctica* (CALB) demonstrated that several factors, such as the order of esters (mono-ester, di-ester or tri-ester), fatty acid ester tail (short chain, long chain, saturated, and unsaturated fatty acids), hydrophilic head groups (sorbitan or isosorbide) and types of enzymes, impact polysorbate hydrolysis (6, 28–30). Therefore, differences observed in the rate of PS20 hydrolysis by lipase and esterase could be due to fatty acid composition in PS20, pH of formulations, and substrate specificity of esterase and lipase in our study.

While FFA levels at 4°C were maintained at the highest levels for up to 3 weeks in both formulations, FFA levels continuously decreased during storage at 37°C. Several factors, such as residual peroxides, traces of residual metal contaminants, temperature, and light impact the auto-oxidation of PS20 (6). Therefore, we hypothesized that FFAs may undergo auto-oxidation at 37°C and that the colorimetric NEFA kit used in our study may not be compatible for detecting oxidized FFAs. There were no data from the manufacturer to support the compatibility of NEFA kit for oxidized fatty acids. We incubated oleic acid, the standard used in the NEFA kit, at 37°C with and without ascorbic acid and copper ions to induce oxidative stress. Metal-catalyzed oxidative stress is known to oxidize fatty acids and our hypothesis for oxidation of FFAs by ascorbic acid and copper ions is based on literature reports, although we did not analytically confirm fatty acid oxidation in our experiments (31). We observed a time-dependent decrease in oleic acid concentration at 37°C compared to its concentration at 4°C, indicating that the decrease in FFA levels detected at 37°C in bevacizumab and T-DM1 formulations could be due to the inability of the NEFA kit to detect oxidized FFAs (supplementary Figure S3). A recent paper by Zhang et al., shows that incubation of fatty acids in glass vials and plastic tubes shows decrease in fatty acid concentration over time at 37°C but this loss of fatty acid concentration is significantly reduced when glass vials are used compared to plastic tubes (32). This study did not compare fatty acid concentration at 4°C and 37°C. We note that all of our experiments were conducted in plastic tubes at both 4°C and 37°C. The use of glass vials could be hypothesized to reduce the relative loss of free fatty acid concentration at 37°C compared to plastic tubes but based on previous study, it may not completely prevent the loss of fatty acid concentration. Therefore, the observed relative decrease in fatty acid concentrations in our experiments at 37°C compared to 4°C in the plastic tubes should not be impacted. To understand whether FFA levels correlate with the formation of FFA subvisible particles, we analyzed subvisible particles using MFI. We observed differences in the number of subvisible particles when PS20 was treated with esterase or lipase, with esterase hydrolysis producing a greater number of particles than lipase. This is consistent with esterase showing more selectivity toward PS20 hydrolysis compared to lipase used in our study. A recent study demonstrated that the susceptibility of PS20 to the formation of FFA particles is dependent on fatty acid composition and enzyme specificity (6). It has previously been demonstrated that there are differences in ester distribution between different grades of PS20, and different enzymes have been shown to have different selectivity for the mono-, di-, and tri-ester forms of polysorbates (6, 28). Because of the heterogeneity of fatty acid composition in different grades of PS20, the solubility of FFAs released by different enzymes under varying conditions could be different. Therefore, differences observed in FFA particle formation between PS20 NF and PS20 PLA in our study are due to the different specificities of esterase and lipase for PS20 NF and PS20 PLA.

Consistent with the higher levels of FFA at 4°C, we observed a greater number of subvisible particles at 4°C compared to 37°C. The greater number of particles recorded in the T-DM1 formulation at pH 5.0 compared to the bevacizumab formulation at pH 6.2 indicates that there is a high risk of formation of FFA particles at acidic pH (low pH) compared to less acidic pH or near neutral pH if hydrolysis of PS20 occurs in the drug formulation during storage. In addition to enzymatic hydrolysis, PS20 is also degraded by chemical hydrolysis at acidic pH (33, 34). Therefore, the formation of a greater number of FFA particles in the T-DM1 formulation at pH 5.0 compared to the bevacizumab formulation at pH 6.2 is due to the increased chemical hydrolysis of PS20 and poor solubility of FFA at low pH. The impact of pH on the release of FFA from PS20 and the formation of FFA particles is further supported by our data collected at pH 5.6 to 7.6. There are no systematic studies to evaluate the impact of pH on the formation of FFA particles in drug formulations, but several studies have reported the formation of subvisible particles in PS20-containing formulations at pH 4.0—5.5 after 2–3 years of storage at 4°C (20, 35). Our study shows that pH is one of the critical factors for assessing FFA particles during formulation development and stability studies when PS20 is used in drug formulation.

Therapeutic protein drugs are generally diluted in suitable diluents and administered within a specified time of storage at room temperature or 4°C based on established in-use stability data for specific drugs, as indicated in the package insert (36, 37). Our data indicate that PS20 NF-containing formulation showed a significant reduction in subvisible particles when warmed from 4°C to room temperature for 2 h and had a lower risk of FFA particle formation than the PS20 PLA-containing formulations. While PS20 PLA contains a relatively high percentage of lauric acid (99.6% C12), the composition of the saturated fatty acids varies lot-to-lot within the wide range of predefined specifications (C12: 40–60%, C14: 14–25%, and C16: 7–15%) in PS20 NF (4, 6). The solubility and critical micelle concentration (CMC) of FFAs depends on the number of backbone carbon atoms and the number of carbon‒carbon double bonds, with lower solubility for longer chain saturated fatty acids compared to shorter chain or unsaturated fatty acids (21, 38–40). Therefore, the differences observed in the solubility of FFA particles at room temperature after dilution in saline could be due to differences in fatty acid composition between PS20 NF and PS20 PLA and the solubility of fatty acids in aqueous solution.

PS20 and PS80 are known to protect monoclonal antibodies against agitation-induced aggregation. Both polysorbates bind to monoclonal antibodies during formulation, but such binding does not alter the secondary structures of antibodies (41). Degradation of PS20 in formulations during storage may impact the stability of therapeutic proteins. A redshift in the trough from 219 to 223 nm and spectral differences at 70°C after lipase or esterase treatment compared to the control suggests some impact on the secondary structures of these therapeutic proteins, resulting in a decrease in stability after PS20 hydrolysis. This change, however, had no impact on the overall integrity and biological activity of bevacizumab and T-DM1 (Fig. 10A and B). A similar effect was observed for PS80 or PS20 degradation, where hydrolysis of polysorbate did not impact the protein stability and biological activity of monoclonal antibodies and recombinant human granulocyte colony stimulating factor (5, 19). Our study was limited to the hydrolytic degradation of PS20 without exposing proteins to mechanical stresses. Mechanical stressors during the handling, storage, and shipping of drugs, such as shaking and dropping, can impact protein stability and functions (42, 43). It is possible that complete hydrolysis of PS20 did not occur under our experimental conditions and that residual amounts of unhydrolyzed PS20 may have been able to protect therapeutic proteins under our experimental conditions. It should be noted that each therapeutic protein has unique physiochemical properties, and our study is limited to only two representative therapeutic proteins in their formulations and pH. A systematic study may be needed using a broad range of therapeutic proteins (antibody isotypes, antibody‒drug conjugates, enzymes, and proteins with different sizes) in a wide range of pH values and formulation excipients using the most common HCP enzymes present in therapeutic proteins for PS20 hydrolysis to understand the product-specific impact of PS20 hydrolysis on product quality.

## Conclusions

Polysorbate degradation and the formation of FFA particles in drug formulations has been a growing concern for the use of polysorbates as surfactants in the pharmaceutical industry. In this work, we compared the hydrolytic degradation of PS20 NF and PS20 PLA using different formulations, enzymes, pH values, and storage conditions for the formation of FFA/FFA particles and the potential impact on the quality of therapeutic proteins. Our results suggest that the fatty acid composition in PS20, particularly % lauric acid (99.6% in PS20 PLA compared to 52.2% in PS20NF used in our study), type of residual hydrolytic enzymes present in the drug formulation, pH, and storage conditions impact PS20 degradation and the formation of subvisible particles. Overall, our results indicate that hydrolysis of polysorbate is one of the sources of the formation of subvisible particles in therapeutic protein formulations, but hydrolysis of PS20 showed no significant impact on protein quality. Our study is limited to two formulations and does not differentiate between FFA particles and proteinaceous particles. Our study is also limited to the measurement of FFA using colorimetric assay and we did not confirm the identity of the released FFA from PS20 hydrolysis using advanced analytical techniques such as mass spectrometry. Additional studies are needed to characterize and better understand the potential safety risk of these FFA particles.

### Supplementary Information

Below is the link to the electronic supplementary material.

Supplementary file1 (PPTX 364 kb)

## Abbreviations

ADCC: Antibody-dependent cellular toxicity
ChP: Chinese Pharmacopoeia
CMC: Critical micelle concentration
EP: European Pharmacopoeia
FFA: Free fatty acid
MFI: Microflow imaging
NF: National formulary
POE: Polyoxyethylene
PS20: Polysorbate 20
PS80: Polysorbate 80
PLA: Percent lauric acid
SDS‒PAGE: Sodium dodecyl sulfate‒polyacrylamide gel electrophoresis
SE-UPLC: Size exclusion ultra-performance liquid chromatography
T-DM1: Trastuzumab emtansine
USP-NF: United States Pharmacopeia and National Formulary
