# Supplementary material for: Stress-mediated polysorbate 20 degradation and its potential impact on therapeutic proteins
Source: Pharm Res. 2024 May 13;41(6):1217–32. doi: 10.1007/s11095-024-03700-7 (PMC11196320; doi:10.1007/s11095-024-03700-7)
Supplement: Supplementary file 1 — Supplementary file1 (PPTX 364 kb) [file 11095_2024_3700_MOESM1_ESM.pptx]

## Slide 1
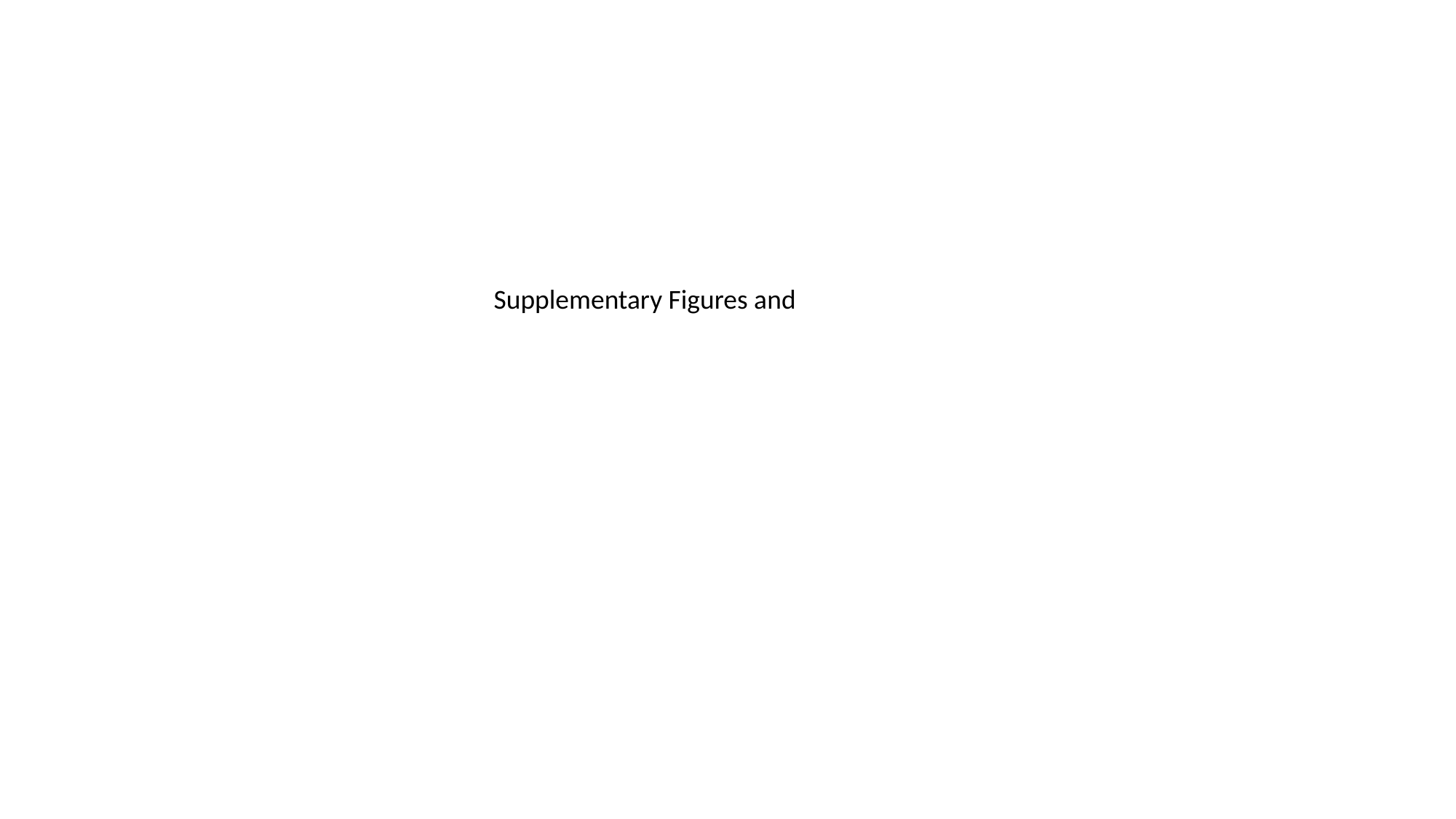

Supplementary Figures and

## Slide 2
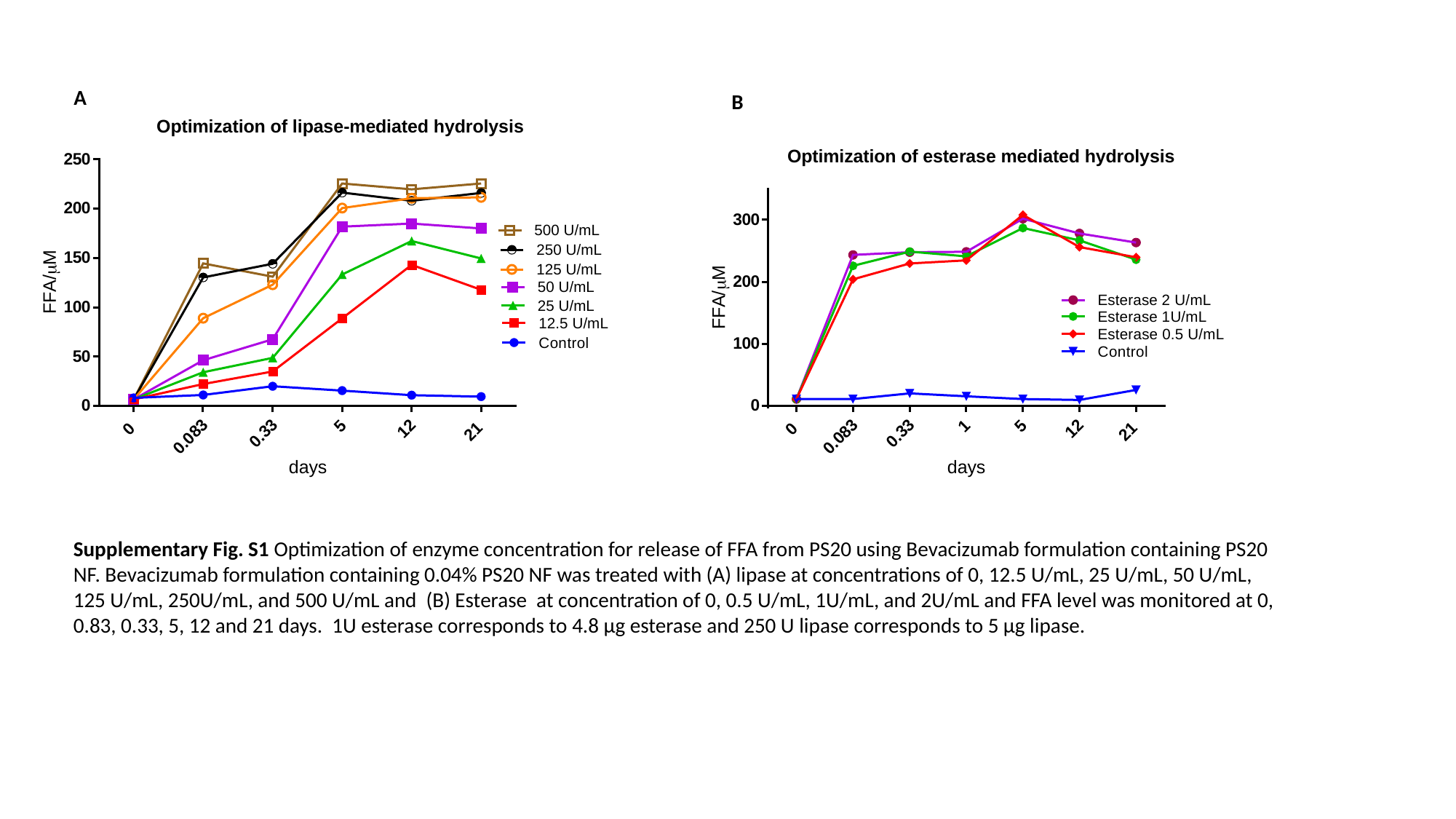

A
B
Supplementary Fig. S1 Optimization of enzyme concentration for release of FFA from PS20 using Bevacizumab formulation containing PS20 NF. Bevacizumab formulation containing 0.04% PS20 NF was treated with (A) lipase at concentrations of 0, 12.5 U/mL, 25 U/mL, 50 U/mL, 125 U/mL, 250U/mL, and 500 U/mL and (B) Esterase at concentration of 0, 0.5 U/mL, 1U/mL, and 2U/mL and FFA level was monitored at 0, 0.83, 0.33, 5, 12 and 21 days. 1U esterase corresponds to 4.8 µg esterase and 250 U lipase corresponds to 5 µg lipase.

## Slide 3
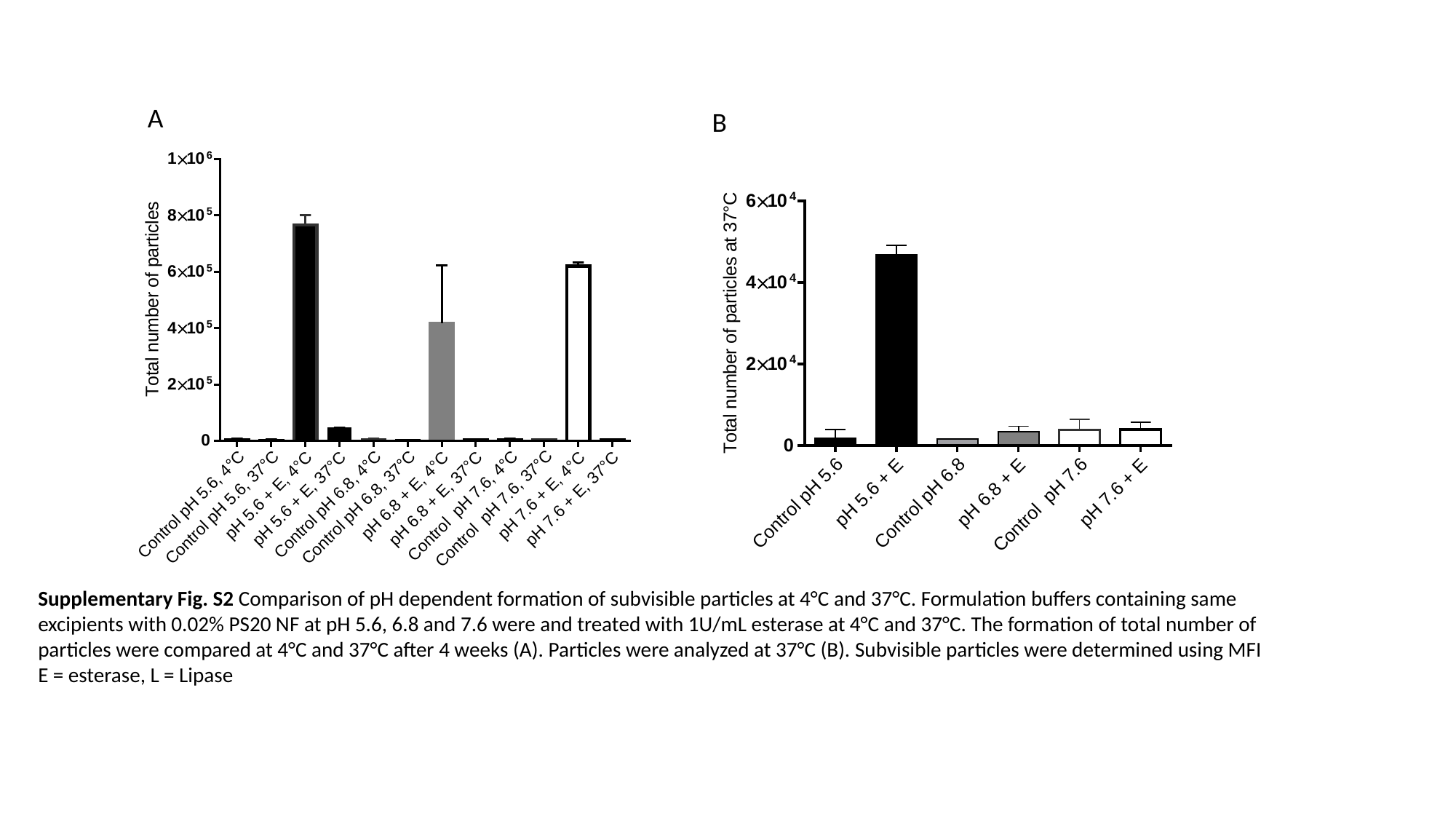

A
B
Supplementary Fig. S2 Comparison of pH dependent formation of subvisible particles at 4°C and 37°C. Formulation buffers containing same excipients with 0.02% PS20 NF at pH 5.6, 6.8 and 7.6 were and treated with 1U/mL esterase at 4°C and 37°C. The formation of total number of particles were compared at 4°C and 37°C after 4 weeks (A). Particles were analyzed at 37°C (B). Subvisible particles were determined using MFI E = esterase, L = Lipase

## Slide 4
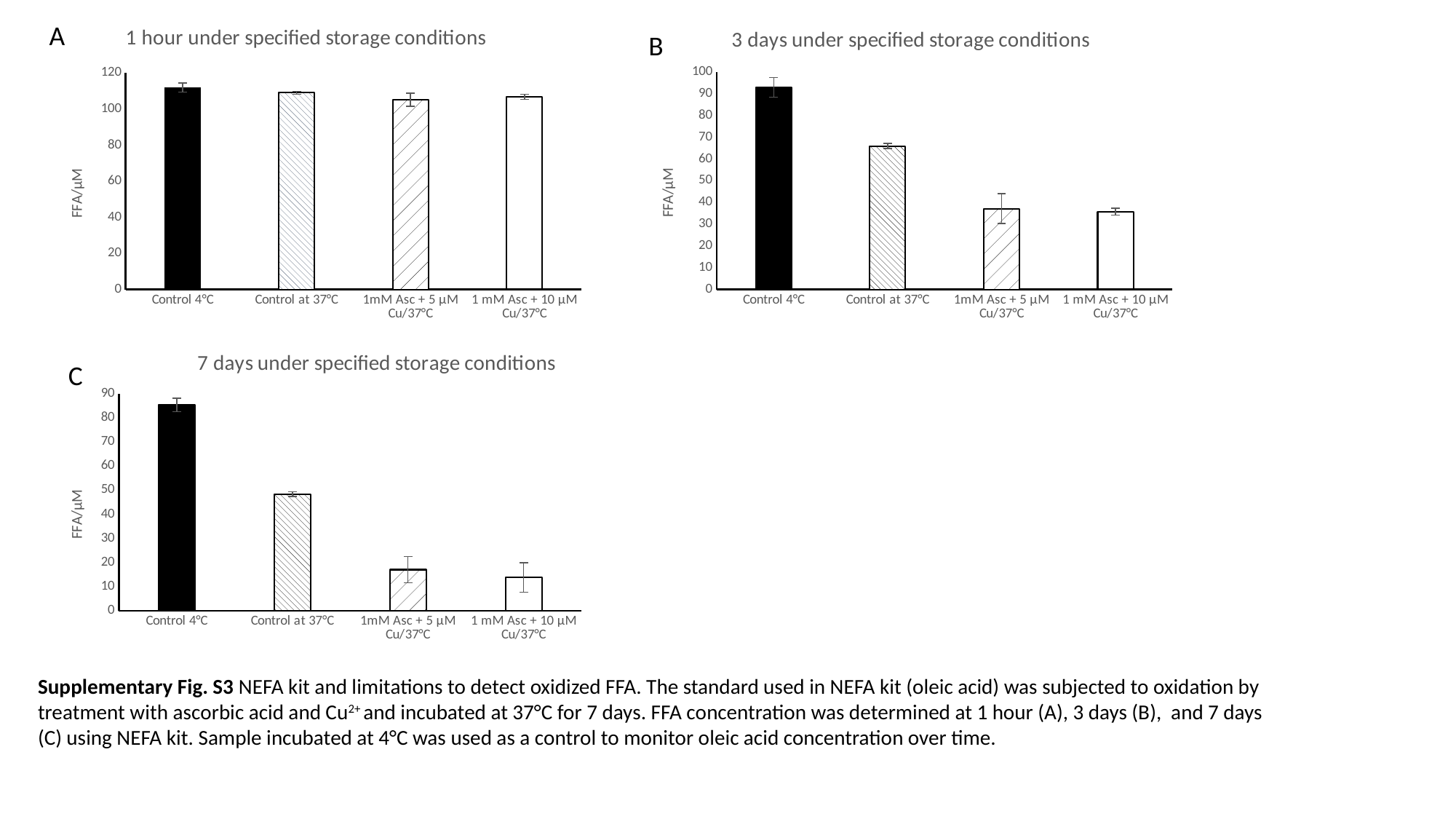

A
### Chart: 3 days under specified storage conditions
| Category | |
|---|---|
| Control 4°C | 93.01960784313728 |
| Control at 37°C | 66.00980392156863 |
| 1mM Asc + 5 µM Cu/37°C | 37.166666666666664 |
| 1 mM Asc + 10 µM Cu/37°C | 35.76470588235295 |
### Chart: 1 hour under specified storage conditions
| Category | |
|---|---|
| Control 4°C | 111.88172043010752 |
| Control at 37°C | 109.05376344086022 |
| 1mM Asc + 5 µM Cu/37°C | 105.21505376344088 |
| 1 mM Asc + 10 µM Cu/37°C | 106.77419354838712 |B
### Chart: 7 days under specified storage conditions
| Category | |
|---|---|
| Control 4°C | 85.21875 |
| Control at 37°C | 48.28124999999999 |
| 1mM Asc + 5 µM Cu/37°C | 17.041666666666668 |
| 1 mM Asc + 10 µM Cu/37°C | 13.822916666666666 |C
Supplementary Fig. S3 NEFA kit and limitations to detect oxidized FFA. The standard used in NEFA kit (oleic acid) was subjected to oxidation by treatment with ascorbic acid and Cu2+ and incubated at 37°C for 7 days. FFA concentration was determined at 1 hour (A), 3 days (B), and 7 days (C) using NEFA kit. Sample incubated at 4°C was used as a control to monitor oleic acid concentration over time.

## Slide 5
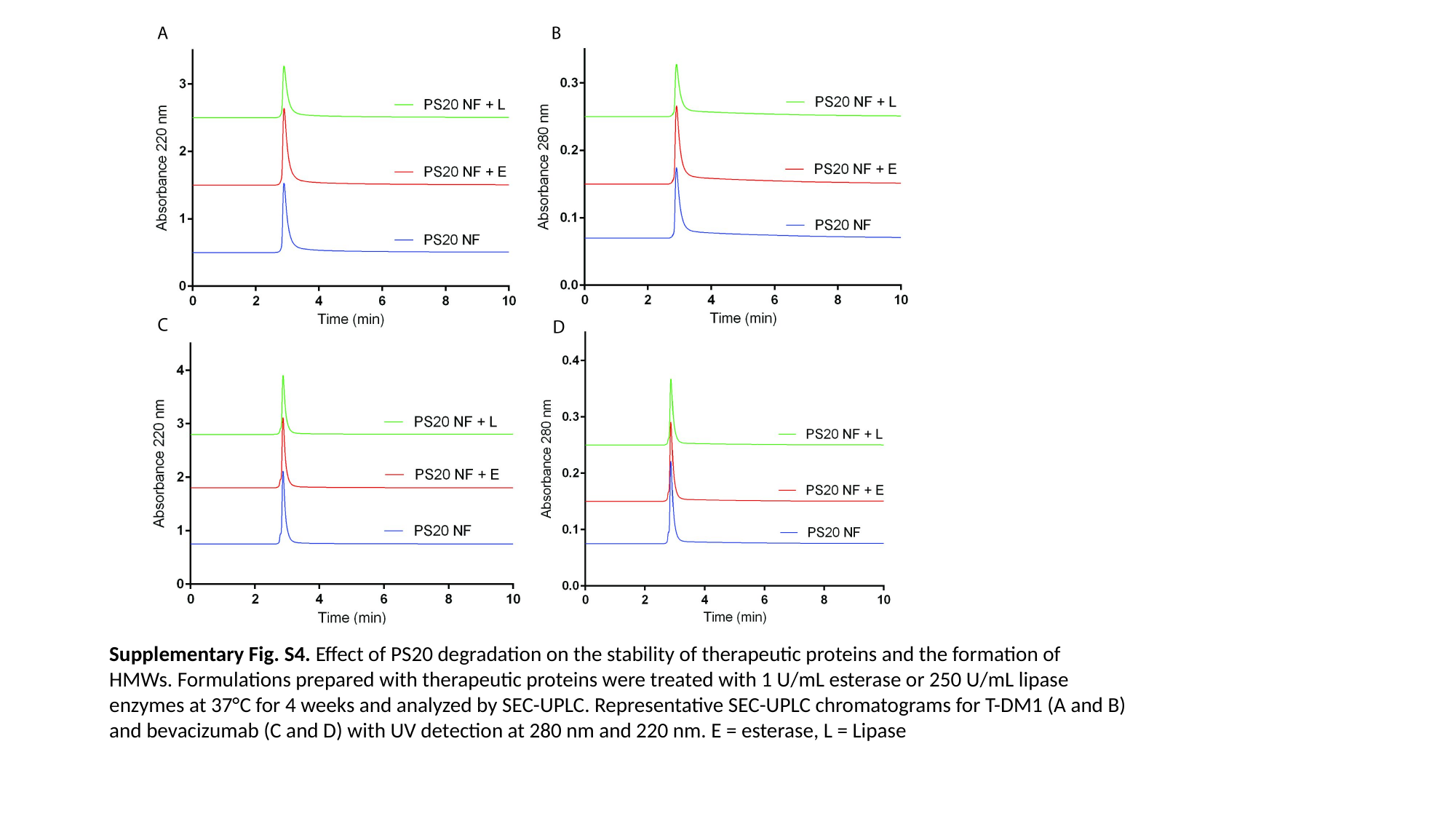

Supplementary Fig. S4. Effect of PS20 degradation on the stability of therapeutic proteins and the formation of HMWs. Formulations prepared with therapeutic proteins were treated with 1 U/mL esterase or 250 U/mL lipase enzymes at 37°C for 4 weeks and analyzed by SEC-UPLC. Representative SEC-UPLC chromatograms for T-DM1 (A and B) and bevacizumab (C and D) with UV detection at 280 nm and 220 nm. E = esterase, L = Lipase

## Slide 6
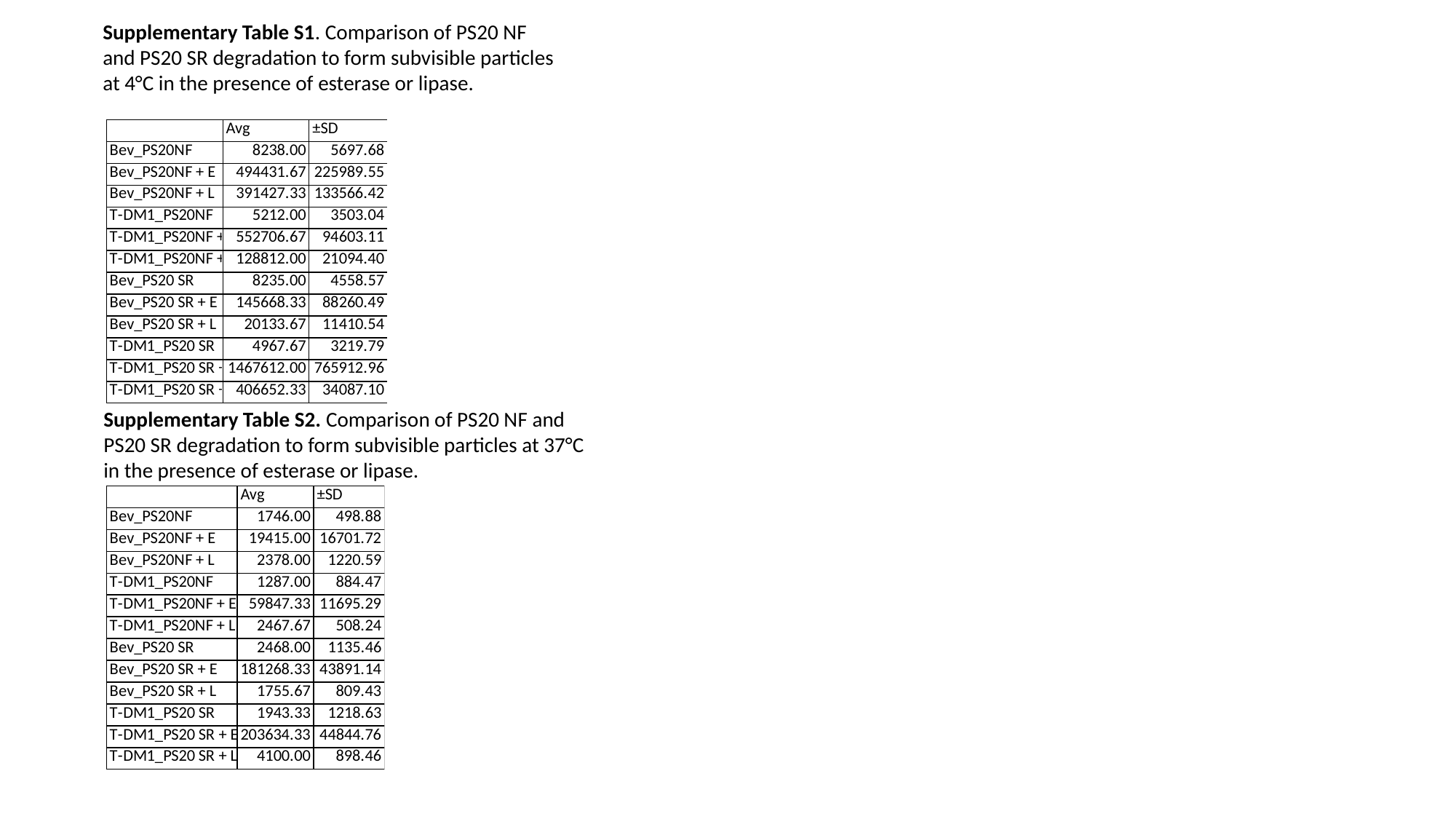

Supplementary Table S1. Comparison of PS20 NF and PS20 SR degradation to form subvisible particles at 4°C in the presence of esterase or lipase.
Supplementary Table S2. Comparison of PS20 NF and PS20 SR degradation to form subvisible particles at 37°C in the presence of esterase or lipase.
